# Age-Dependent Transcriptome and Proteome Following Transection of Neonatal Spinal Cord of *Monodelphis domestica* (South American Grey Short-Tailed Opossum)

**DOI:** 10.1371/journal.pone.0099080

**Published:** 2014-06-10

**Authors:** Norman R. Saunders, Natassya M. Noor, Katarzyna M. Dziegielewska, Benjamin J. Wheaton, Shane A. Liddelow, David L. Steer, C. Joakim Ek, Mark D. Habgood, Matthew J. Wakefield, Helen Lindsay, Jessie Truettner, Robert D. Miller, A. Ian Smith, W. Dalton Dietrich

**Affiliations:** 1 Department of Pharmacology & Therapeutics, The University of Melbourne, Victoria, Australia; 2 Department of Neurobiology, Stanford University, Stanford, California, United States of America; 3 Department of Biochemistry and Molecular Biology, Monash University, Clayton, Victoria, Australia; 4 Department of Neuroscience and Physiology, University of Gothenburg, Gothenburg, Sweden; 5 Walter & Eliza Hall Institute of Medical Research, Victoria, Australia; 6 Department of Genetics, The University of Melbourne, Victoria, Australia; 7 Institute of Molecular Life Sciences, University of Zurich, Zurich, Switzerland; 8 The Miami Project to Cure Paralysis, University of Miami, Miller School of Medicine, Miami, Florida, United States of America; 9 Center for Evolutionary & Theoretical Immunology, Department of Biology, University of New Mexico, Albuquerque, New Mexico, United States of America; CSIRO, Australia

## Abstract

This study describes a combined transcriptome and proteome analysis of *Monodelphis domestica* response to spinal cord injury at two different postnatal ages. Previously we showed that complete transection at postnatal day 7 (P7) is followed by profuse axon growth across the lesion with near-normal locomotion and swimming when adult. In contrast, at P28 there is no axon growth across the lesion, the animals exhibit weight-bearing locomotion, but cannot use hind limbs when swimming. Here we examined changes in gene and protein expression in the segment of spinal cord rostral to the lesion at 24 h after transection at P7 and at P28. Following injury at P7 only forty genes changed (all increased expression); most were immune/inflammatory genes. Following injury at P28 many more genes changed their expression and the magnitude of change for some genes was strikingly greater. Again many were associated with the immune/inflammation response. In functional groups known to be inhibitory to regeneration in adult cords the expression changes were generally muted, in some cases opposite to that required to account for neurite inhibition. For example myelin basic protein expression was reduced following injury at P28 both at the gene and protein levels. Only four genes from families with extracellular matrix functions thought to influence neurite outgrowth in adult injured cords showed substantial changes in expression following injury at P28: Olfactomedin 4 (*Olfm4*, 480 fold compared to controls), matrix metallopeptidase (*Mmp1*, 104 fold), papilin (*Papln*, 152 fold) and integrin α4 (*Itga4*, 57 fold). These data provide a resource for investigation of *a priori* hypotheses in future studies of mechanisms of spinal cord regeneration in immature animals compared to lack of regeneration at more mature stages.

## Introduction

↑The studies of Aguayo and colleagues in the 1980s [1], [2], which repeated an old experiment of Tello [3] using implants and bridges of sciatic nerve to promote regeneration of the central nervous system (CNS) resulted in a concentrated effort to understand the mechanisms underlying the failure of the adult mammalian spinal cord to exhibit regenerative recovery following injury. Since then several such inhibitory mechanisms have been described including myelin inhibitory factors [4], [5] and proteoglycans [6]–[8]. So far no effective therapy has emerged and disappointingly, attempts to replicate apparently promising animal studies have been mostly unsuccessful (e.g., [9]–[13]). One reason for this may simply be that the responses of the injured CNS are so complex that a repair strategy based on modifying only one aspect of this process is unlikely to be successful. A strong indication of the complexity of the response of the spinal cord to injury came first from the microarray study by Aimone and colleagues [14]. Similarly complex findings following brain injury have been reported [15]. Verhaagen et al. [16] provided an overview of 25 gene expression profiling studies (over the period of 2001 to 2009, see [16]) of spinal cord injury in rodents. The sites examined were at or around the lesion, which were mainly a contusion in the thoracic spinal cord. The studies used microarrays (generally Affymetrix) to investigate gene expression over a wide range of post injury times (30 min to 90 days). One of these studies involved embryonic spinal cord, which was uninjured but compared to adult injured spinal cord [17]. To date, only one RNA-Seq dataset of injured spinal cord has been published [18] although additional information may perhaps be gleaned from an RNA-Seq study that examined the effects of transplanted progenitor/stem cells following spinal cord injury [19].

Verhaagen et al. [16] reported that the observed changes in gene expression across this large number of studies were “remarkably consistent” and summarized the results under 8 functional categories that showed changed expression. Genes in some of these groups were generally upregulated: immediate early genes, proinflammatory genes, phagocytosis & induction of the complement system, neuronal genes (some implicated in neurite outgrowth or synaptic plasticity). Other groups showed a mixture of up- and downregulation: genes related to apoptosis, angiogenesis. Some showed predominantly downregulation: genes encoding neurotransmitter synthesis and other aspects of synaptic function, ion channels. Surprisingly, no mention was made of genes that generate various extracellular matrix factors such as proteoglycans, as this family of proteins has been implicated in several studies to be involved in the failure of neurite outgrowth following injury (see [20]).

In contrast to the lack of functional recovery from severe spinal cord injury in adult mammals, immature animals show a significant degree of recovery. This has been demonstrated in two species of marsupial opossum, where substantial growth of axons across a lesion was observed. Some of these axons were shown to be regenerating and some were growing as a part of normal development [21]–[23]. This response to injury appears to be age dependent [23]–[25] providing an opportunity to compare gene regulation in a mammalian species at a stage of development when regeneration and axon growth occurs and one when it does not [25], [26]. To undertake a similar study in rodents would require *in utero* transection of the spinal cord at around E15 (permissive stage, [27]) and at a stage that was non-permissive but with bodyweight bearing locomotion (probably E19–20).

Some preliminary studies in developing spinal cord have been carried out using human microarrays [28], mouse microarrays [24] and tammar wallaby microarrays [29] as well as different forms of polymerase chain reaction methods [24], [30]. These showed that there is indeed substantial age-dependent gene regulation in the response of the spinal cord to injury [24], [28]–[30]. The most comprehensive study of these responses was that by Mladinic et al., [29] using a combination of microarray (tammar wallaby, a marsupial species) and qRT-PCR for specific genes of interest. These authors divided the genes identified into categories relating to whether changes in expression (up- or downregulation) might be expected to be contributing to successful growth of axons across a lesion made at P8, but not at P13. However, these studies used *in vitro* preparations of isolated *Monodelphis* spinal cord at 25°C, which may have had an influence on some aspects of the response to injury (see General Discussion).

With the recent sequencing of the opossum genome [31] and the advent of high throughput RNA sequencing (RNA-Seq) it is now possible to examine overall gene expression changes in response to injury in this species. In the present study we investigated both the transcriptome and the proteome in the segment of cord rostral to the site of injury following a complete spinal cord transection in opossums at an age when axonal growth across a lesion and substantially normal locomotor development occur (postnatal day 7, P7) and compared this with an older age when no axon growth can be seen, but nevertheless a demonstrable body weight-bearing locomotion is present (P28, [25]). A study of changes in the spinal cord proteome caudal to the site of injury following spinal cord transection in *Monodelphis* at these two ages has been previously published [32].

## Materials and Methods

### Animals used

*Monodelphis domestica* were obtained from a colony based at the University of Melbourne Medical Sciences Animal House Facility, Melbourne, Australia. Procedures were performed according to National Health and Medical Research Council guidelines, with the approval of the University of Melbourne Animal Ethics Committee (Ethics #0707108). Pups of both sexes were used. Day of birth was designated as postnatal day zero, P0 (see [25], [26]).

The pups were assigned to two age groups and spinal cord injuries (SCIs) were performed at either P7 or P28. At P7, whole litters (6–7 pups) were operated on. Separate litters of pups were kept as controls as there is no consistent way to mark these very young animals without increasing the risk of cannibalisation by the mother [22]. Injuries at P28 were usually made on half the pups in a litter, since at P28 their ears can be marked. The remaining pups from these litters were anaesthetised but remained uninjured and were used as controls. For RNA-Seq and proteomic analyses experimental and control pups were collected at 24 h (+24 h) post surgery. For morphological studies, animals were collected as unoperated controls and at 0 h and 24 h post injury in both age groups. The total number of animals used in the transcriptomic study was: P8 control (*n* = 24), P7+24 h (*n* = 24), P29 control (*n* = 12), P28+24 h (*n* = 12). These were obtained from several separate litters. In the proteomic study the number of animals and weights of tissues used are shown in Table 1. For the morphological studies at least 3 pups at each age were used, usually from different litters [32], [33].

**Table 1:** Number, tissue weight and protein concentration of spinal cord tissue used for proteomic analysis in this study.

Age group	Number of cords	Tissue weight (mg)	Total protein concentration (µg/µl)
**P7+24 h transected**	11	37.7	4.7
**P8 control**	11	27.8	5.14
**P28+24 h transected**	4	66.5	6.97
**P29 control**	4	87	8.74

Samples rostral to the site of injury (T10) were used in proteomic analysis for control and transected spinal cords of *Monodelphis domestica* at P7 or P28. Note that individual cords were pooled from more than one litter.

### Spinal cord transection

At P7, *Monodelphis* pups are still attached to the mothers' teats [34]. The female adult *Monodelphis* were anaesthetized with 2–3% isofluorane; the same anaesthetic was administered to the P7 pups via a small facemask during the surgical procedure. Pups at P28 are no longer attached to the mother and were separately anaesthetized with isofluorane throughout the surgical procedure [25].

Complete spinal cord transection was performed at thoracic level 10 (T10) using sharp sterilized fine scissors. Skin was closed using surgical grade glue (Vetbond, 3 M, St. Paul, MN, USA). Animals were returned to their cages and allowed to recover for 24 hours (+24 h) post injury. At the end of the experimental period, control and injured animals were terminally anaesthetized with an overdose of isofluorane and spinal cords were dissected out.

The cords were separated into two segments, the upper (rostral to the injury) and lower (caudal to the injury) divided at T10 (site of transection), or corresponding segments from control animal spinal cords. Samples were stored at −80°C until used. Rostral segments of the cords were used in the present study. For morphological studies spinal cords were dissected out, fixed in Bouin's fixative and paraffin embedded as described previously [32], [33].

### RNA extraction

Samples of rostral cord were homogenized using Qiashredder columns (Qiagen, Valencia, CA, USA) and total RNA was extracted using the RNeasy Plus Mini Kit (Qiagen) according to standard supplier protocol. Total RNA samples were quantified using a NanoDrop ND-100 UV-VIS spectrophotometer (Thermo Scientific, Wilmington, DE, USA) and quality checked on an RNA chip using and Agilent 2100 Bioanalyzer (Agilent, Santa Clara, CA, USA). Only samples with an RNA Integrity Number close to 9 were kept for further sequencing experiments.

### RNA sequencing

RNA sequencing was performed at the Australian Genome Research Facility (Melbourne, Australia). A cDNA library was prepared from 10 µg of total RNA from pools of two individuals using the RNA-Seq Sample Preparation Kit (Illumina, San Diego, CA, USA) according to the standard manufacturer protocol. Quality of the library was verified using a DNA 1000 chip using the Agilent 2100 Bioanalyzer (Agilent) and quantified by fluorimetry. The library was subjected to 100 bp single end read cycles of sequencing on an Illumina Genome Analyzer IIx (Illumina) as per manufacturer protocol. Cluster generation was performed on a c-Bot (Illumina) with a single read cluster generation kit. Sequencing was performed using a 36-cycle sequencing kit v4. In total 16 separate sequencing lanes were run on the platform. Two separate runs were conducted from separately collected samples, which were: rostral spinal cords from P8 control, P7+24 h injury, P29 controls and P28+24 h injury.

### Statistical analysis of RNA-Seq data

#### Gene expression level analysis

Short reads were trimmed to remove ambiguous bases from the start and segments with low quality scores from the end. Trimmed reads were mapped with Bowtie2 version 2.0.4 [35] to the Ensembl *Monodelphis domestica* genome, release 69 [36]. The number of reads mapped to nuclear genes was determined with HTSeq [37], using the default “union” counting option.

An average of 4.6 M mapped reads were obtained per sample. Raw data are available at: Gene Expression Omnibus (http://www.ncbi.nlm.nih.gov/geo/) under accession code GSE54805. Differential expression between the adult and embryonic samples was detected using an exact test in the Bioconductor [38] 6dger package version 2.6.12 [39]. Genes considered to be significantly differentially expressed were those with a *p*-value of less than 0.05 after false discovery rate correction. Changes in expression were considered significant where there was a fold change greater than 2.00 and an adjusted *p*-value of less than 0.05. Gene targets with fold changes less than 2.00 were considered unchanged. Genes that showed changes according to the above criteria are shown Table S1 (P7+24 h) and Table S2 (P28+24 h).

#### Gene ontology level analysis

Gene Ontology (GO) analysis was completed using GOSeq software [40]. Differentially expressed genes were split into up- and down-regulated groups and a separate GOSeq enrichment test was applied for each set. A final Benjamini-Hochberg correction was applied to adjust for multiple tests.

Illumina RNA sequencing data have been deposited with the Gene Expression Omnibus http://www.ncbi.nlm.nih.gov/geo/) under accession code GSE54805.

### Proteomics

Methods for the 2-dimensional separation of proteins, selection of protein bands and mass spectrometry have been published previously [32] but are described here in full.

#### Sample preparation for proteomic analysis

Spinal cord segments rostral to the site of injury performed at P7 or P28 were collected 24 hours after transection (P7+24 h or P28+24 h) together with corresponding segments from age-matched controls. Tissue was pooled from several pups (n = 4–20) in order to obtain a total of a minimum of 30 mg (wet tissue weight) per sample (Table 1). Pooled tissue samples were homogenized 1∶10 w/v in homogenization buffer containing 0.32 mM sucrose, 25 mM Tris, 1 mM MgCl2, pH 7. This was done by passing the samples through 20 Gauge (G), 21 G, 25 G and 27 G needles until the suspension offered no more resistance. Samples were centrifuged at 2000×*g* for 2 minutes at 4°C and supernatants retained for further analysis. Total protein concentration was estimated using the Bradford Assay [40] with a protein standard (Sigma-Aldrich, St Louis, MO, USA) to ensure the consistency of the extraction process as all samples were normalized weight to volume. The same volume for all samples was used throughout the study (Table 1).

#### The clean-up step

Contaminants were removed from 50 µl aliquots of each sample using the 2-dimensional (2D) clean-up kit (GE Healthcare Bio-Sciences Corp., Piscataway, NJ, USA) as detailed in the Manufacturer's Protocol (Procedure B). Fifty µl aliquots of each sample were used for the clean-up. Samples were centrifuged for 10 minutes at 8000×*g* and wash buffer was removed without disturbing the pellet. Acetone present in the wash buffer was evaporated before moving to the next step. Six clean-up samples were prepared for each age group,

#### Off-gel Fractionator

An Off-gel Fractionator 3100 (Agilent Technologies, Santa Clara, CA, USA) was used in accordance with the Manufacturer's Protocol. Immobilized pH gradient (IPG) strips (12 cm, pH 3–10, Linear, Agilent Technologies, Santa Clara, CA, USA) were used for this separation. A total of 150 µl from cleaned-up sample (see above) was rehydrated (as specified by Manufacturer's Protocol) and prepared for each lane together with IPG strips (also rehydrated with the Off-gel buffer prior to sample loading). Each sample was run in duplicate on two separate IEF lanes. The fractionator was set to run under the default Manufacturer's settings until the current was reduced to zero. Samples from each well were collected and the Bradford assay [40] was performed on each sample. Variation in protein concentrations between the duplicates was within ±10%. For further analysis duplicates of each sample fraction were combined and all Off-gel fractions were used. Aliquots (25 µl) from each fraction were subjected to a further clean-up step as described above. Again, duplicates were prepared for each sample. In the final step, the wash buffer was carefully decanted without disturbing the pellet before pellets were dried at 37.5°C in a heat block for 10 minutes to fully evaporate any wash buffer residue.

#### Lithium dodecyl sulfate –polyacrylamide gel electrophoresis (LDS-PAGE)

The obtained dried pellet was re-suspended in 5 µl LDS sample buffer (Invitrogen, Carlsbad, CA, USA) [23], 2 µl reducing agent (Invitrogen, Carlsbad, CA, USA) and 13 µl deionized water. The mixture was heated in a 37.5°C heat block for 10 minutes. Pre-cast 4–12% NuPage Bis-Tris 10 well Mini Gels (Invitrogen, Carlsbad, CA, USA) were used with 2-(N-morpholino) ethanesulfonic acid (MES)-SDS running buffer (Invitrogen, Carlsbad, CA, USA) diluted 1∶20. Samples from each fraction from injured and control animals were loaded as duplicates. One lane of each gel contained a molecular weight standard (NovexH Sharp pre-stained standard, Invitrogen, Carlsbad, CA, USA). Gels were run at 200 V constant voltage for approximately 35 minutes.

#### Silver Staining and Densitometric Gel Analysis

Separated protein bands were visualised using silver stain. Gels were stained for 10–15 min (Silver Stain Plus Kit, Bio-Rad Laboratories, Hercules, CA, USA) according to the Manufacturer's Protocol. Silver stained gels were scanned on a flatbed scanner (Agfa Duoscan, Mortsel, Belgium) and analysed using 1D gel analysis software, GeneTools V4.01.02 (Syngene, Synoptics Ltd, Cambridge, England). The number of bands visible in each lane was counted and consistency between duplicates in each lane was checked. Protein profiles from injured spinal cord samples were compared to those from controls for each fraction. Differences in band intensity, staining pattern or molecular weight changes were recorded. A relative change threshold of ±0.5 compared to control (set as 1) was accepted to identify proteins that changed their expression following spinal injury. This threshold was set after evaluation of technical variability of the methods employed [32], [41]. As is usually the case for such proteomic studies it was not possible to make a sufficient number of biological replicates for a statistical analysis to be applied. The results are therefore presented as an increase or decrease compared to controls.

### Mass Spectrometry

#### Tryptic digestion

Each protein band of interest was individually and manually excised from gels and de-stained in 50 mM ammonium bicarbonate with 50% acetonitrile. Obtained gel pieces were washed and dehydrated in 50 mM ammonium bicarbonate and acetonitrile in alternating wash steps until completely dehydrated. Once dehydrated, gel pieces were subsequently rehydrated in 0.5 µg trypsin (Promega corp., Madison, WI, USA) and 20 mM ammonium bicarbonate solution for in-gel digestion by incubating at 37°C overnight and sonicated (Health Sonics, Livermore CA, USA) for 10 minutes prior to analysis.

#### LC-MS/MS

Tryptic digests were analysed by LC-MS/MS using the HCT ULTRA ion trap mass spectrometer (Bruker Daltonics, Bremen, Germany) coupled online with nanoflow HPLC (Ultimate 3000, Thermo Scientific, Breman, germany). Samples injected onto a pepmap100, 75 µm id, 100 Å pore size, reversed phase nano column with 95% buffer A (0.1% Formic acid) at a flow rate of 300 nl/minute. The peptides were eluted over a 30-minute gradient to 70% B (80% Acetonitrile 0.1% formic acid). The eluant is nebulised and ionised using the Bruker ESI electrospray source via the nanoflow ESI sprayer with a capillary voltage of 4000 V, dry gas at 200°C and flow rate of 5.0l/minute and nebuliser gas at 6psi. Peptides are selected for MSMS analysis in autoMSn mode with smart parameter settings selected with a target mass of 900 m/z and active exclusion released after 1 minute. Data obtained from LC-MS/MS were searched against a custom database downloaded from the National Center for Biotechnology Information (NCBI) ftp site and Swiss-Prot databases using the MASCOT search engine (version 2.1, Matrix Science Inc., London, UK) with all taxonomy selected.

Identified proteins were categorized by relevance to spinal cord injury, obtained from search of relevant literature published in PubMed (http://www.ncbi.nlm.nih.gov/pubmed).

### Morphology and Immunohistochemistry

Bouin's-fixed, paraffin embedded spinal cords from P8, P7+24 h, P29 and P28+24 h animals (n = 3–4) were obtained from previous studies [32], [33] with some additional material collected specifically for this project. All sections were cut in either coronal or sagittal plane at 5 µm thickness. Ten consecutive sections were placed on each glass slide. Routine hematoxylin and eosin (H&E) staining was performed on every 10^th^ slide for general morphology. Immunocytochemistry using the PAP (peroxidase-anti-peroxidase) detection method [32], [42] was applied to map the cellular distribution of individual proteins. Briefly, sections were dewaxed in histolene (Fronine, Australia) followed by rehydration in ethanol of decreasing concentration and final wash in phosphate buffered saline with 0.2% Tween20 (PBS/Tween). After blocking non-specific binding sites with Peroxidase and Protein Blockers (DAKO) sections were incubated with primary antibodies (rabbit anti- human IL-1β, Endogen, USA). This was followed by consecutive incubations with appropriate secondary antibodies (swine anti rabbit, DAKO, 1∶200 dilution and rabbit PAP, Sigma, 1∶200 dilution) and developed with DAKO DAB+ detection kit. Finally the stained sections were dehydrated through graded alcohol and histolene and mounted with Ultramount (Fronine, Australia). Control sections did not contain the primary antibody and these always appeared blank.

The presence of myelin was detected in paraffin sections by the histological stain Luxol Fast Blue as described in detail previously [25].

## Results and Discussion

### General Morphology of the injury site of *Monodelphis* spinal cord injured at P7 or P28

**Figure 1:**
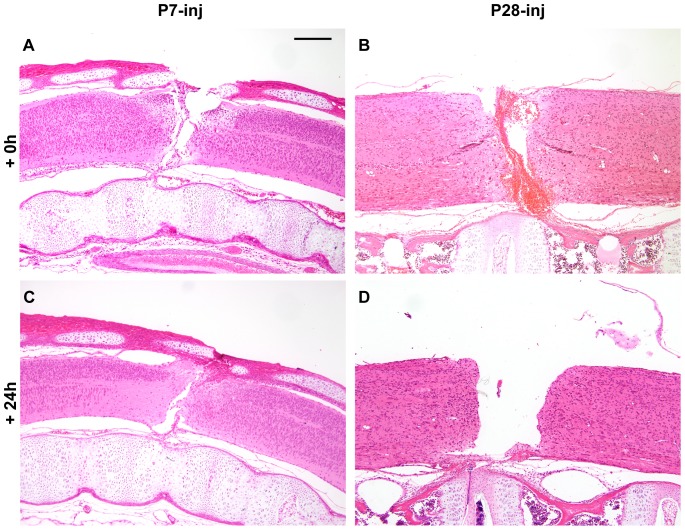
*Monodelphis domestica* spinal cords injured at P7 or P28. Longitudinal sections (hematoxylin & eosin staining) of spinal cords injured at P7 or P28 shown immediately after complete spinal transection at T10 (A, B) or 24 hours later (C, D). Note obvious bleeding into the injury site at P28 (B), which was more pronounced than at P7 (A) One day after transection (+24 h) the gap between severed ends of the cord was larger in P28 injured animals (D) than in P7 injured animals (C). Rostral end is to the left, caudal to the right, dorsal is uppermost. Scale bar is 500 µm.

The morphological appearance of the spinal cord of *Monodelphis* after injury is illustrated in H&E stained sections in Fig. 1. Note the completeness of the transections at P7 and P28 (Fig. 1A&B) and the obvious bleeding into the wound site in the P28 spinal cord (Fig. 1B). It was noticeable that at the time of injury bleeding was more prominent in the P28 spinal cords than at P7. It is likely that bleeding occurred in some of the P7 injured cords collected for RNA-Seq analysis, but the P28 cords were probably more contaminated, which may account for the presence of upregulated blood-related genes at P28 but not P7 following injury (see below). Note also that the gap between the rostral and caudal ends of the transected spinal cord at 24 h post injury was much greater in the P28 (Fig. 1D) cord compared to the P7 cord (Fig. 1C). A similar difference between P7 and P14 transected cords was reported previously [24]. This difference was attributed to greater arching of the back in the P14 *Monodelphis*. The even larger gap in transected spinal cords of older opossum may contribute to the lack of neurite growth across the site of injury in P28 pups. Nevertheless at both ages 24 h after injury the cut ends of the spinal cord were clearly sealed without showing much cellular damage in the surrounding spinal cord tissue.

### Transcriptomic analysis of postnatal *Monodelphis* spinal cord following transection

**Figure 2:**
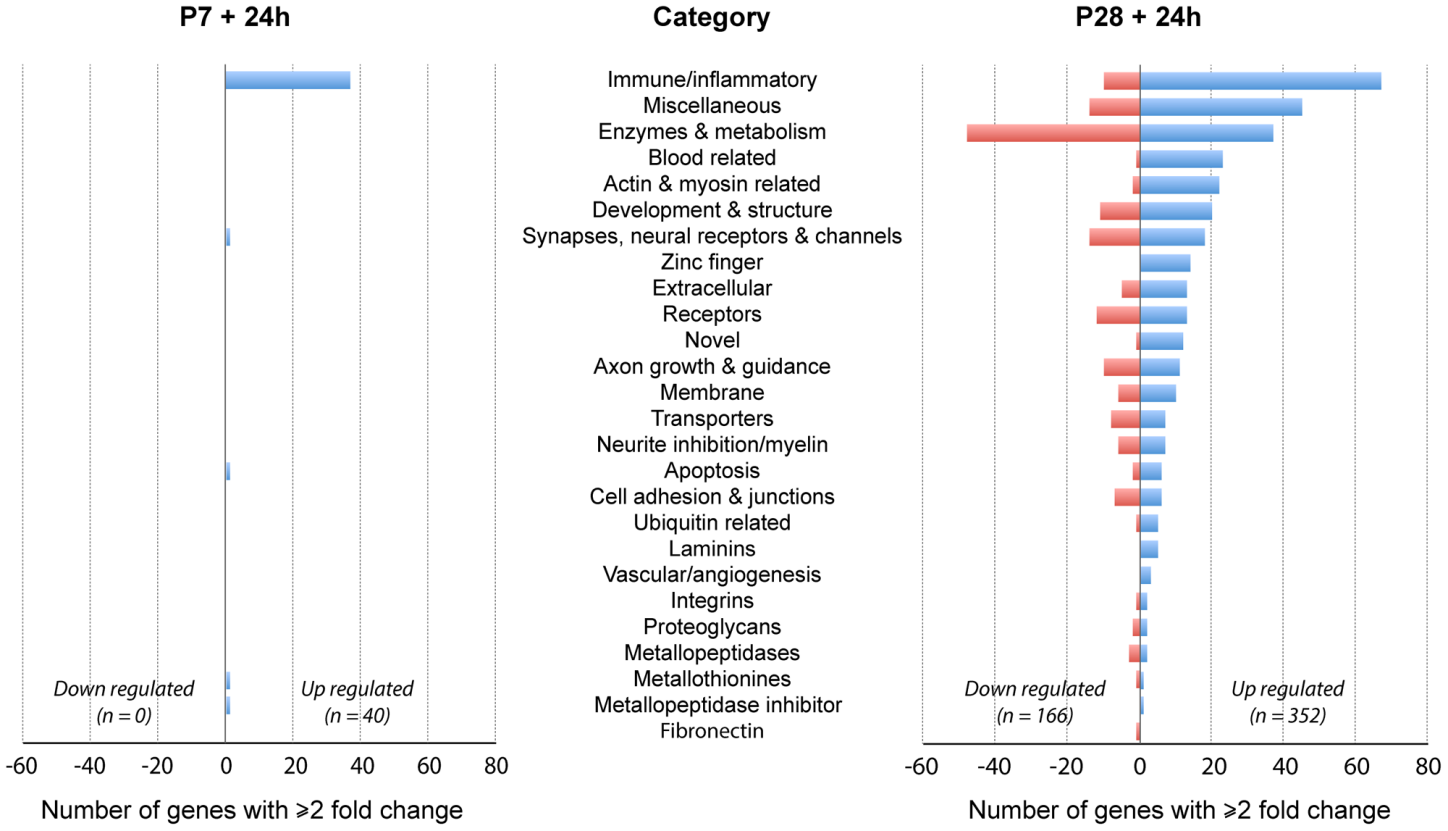
Changes in gene expression in spinal cord 24*Monodelphis domestica*. Numbers of genes in each functional category that showed ±≥2 fold change compared to uninjured aged matched controls. Note dominance of immune/inflammatory genes particularly at P7. Also note that at P28 many more genes showed expression changes (both up and down). See Tables S1 and S2 for gene descriptions, fold change and p values.

The gene expression responses to injury have been examined in the segment of spinal cord rostral to a complete spinal transection (T10) at two postnatal ages: P7 when substantial axon growth occurs across the lesion site and P28 when no such axon growth occurs [25]. Gene expression patterns were investigated using high throughput RNA-Seq analysis at 24 h after injury. Genes were assigned to functional categories based on published information on function, taking account of studies of gene expression changes in injured adult spinal cord (e.g., [16], [17], [20], [43]) as well as those we deduce might have effects on neurite outgrowth at P7 and P28, 24 h after spinal cord transection (Fig. 2 and Tables S2 and S3). Only a single assignment was made for each gene (Tables 2–4, S5), although many appear to have multiple functions.

**Table 2:** Change in expression of neurite inhibitory and axon growth/guidance genes 24 h following spinal cord injury at P28.

SYMBOL	GENE DESCRIPTION	FOLD
	**NEURITE INHIBITION/MYELIN**	
***Efna4***	ephrin-A4	5.0
***Arhgap6***	Rho GTPase activating protein 6	3.1
***Gmip***	GEM interacting protein encodes a member of the ARHGAP family of Rho/Rac/Cdc42-like GTPase activating proteins.	3.0
***Novel***	similar to SMPD3 sphingomyelin phosphodiesterase	2.4
***Novel***	similar to OPHN1 oligophrenin 1,encodes Rho-GTPase-activating protein	2.3
***Mpz***	myelin protein zero	1.8
***Rtn4***	Nogo, myelin inhibitory factor	1.2
***Mog***	myelin oligodendrocyte glycoprotein	−1.7
***Mag***	myelin associated glycoprotein	−1.8
***Arhgef10***	Rho guanine nucleotide exchange factor (GEF) 10	−2.0
***Arhgap35***	Rho GTPase activating protein 35	−2.1
***Mbp***	myelin basic protein	−2.2
***Plp1***	proteolipid protein 1	−2.4
	**AXON GROWTH & GUIDANCE**	
***Hhipl2***	HHIP-like 2, hedgehog interacting protein-like 2	6.5
***Sgsm3***	small G protein signaling modulator 3	4.5
***Slit3***	slit homolog 3 (Drosophila) interacts with Robo; axon repellent	4.0
***Novel***	similar to DNAH7 dynein, axonemal, heavy chain	3.1
***Robo3***	roundabout, axon guidance receptor, homolog 3 (Drosophila)	3.0
***Mical1***	microtubule assoc monoxygenase, calponin & LIM domain containing 1	2.3
***Serpinf1***	serpin peptidase inhib, clade F α-2 antiplasmin, pigment epithelium factor)	2.3
***Tgfb3***	transforming growth factor, β 3, regulator of ECMs and integrins	2.2
***Ntn5***	netrin 5	2.1
***Tcerg1l***	transcription elongation regulator 1-like	2.0
***Elmo3***	engulfment and cell motility 3	2.0
***Cntn2***	contactin 2 (axonal) [Source:HGNC Symbol;Acc:2172]	−2.0
***Sema4f***	sema domain, immunoglobulin domain (Ig), transmembrane domain (TM)	−2.0
***Astn1***	astrotactin 1, neuronal adhesion molecule required for glial-guided migration of young postmitotic neuroblasts, previously described in developing brain	−2.1
***Olig2***	oligodendrocyte lineage transcrip factor 2, enhances myelination after SCI	−2.1
***Pak7***	p21 protein (Cdc42/Rac)-activated kinase 7	−2.3
***Plekhb1***	pleckstrin homology domain containing, family B (evectins) member 1	−2.4
***Sema5a***	sema domain, seven thrombospondin repeats (type 1 and type 1-like), transmembrane domain TMand short cytoplasmic domain, (semaphorin) 5A	−2.4
***Sema3e***	sema domain, immunoglobulin domain (Ig), short basic domain, secreted	−2.5
***Pak2***	p21 protein (Cdc42/Rac)-activated kinase 2	−2.9
***Gas7***	growth arrest-specific 7, neurite outgrowth in some cultured neurons	−7.7

Change in expression of neurite inhibitory and axon growth/guidance genes in rostral spinal cord 24 h following spinal cord injury at P28. Note that most of the myelin inhibitory factor genes have not changed their expression in response to injury (fold <2).

**Table 3:** Change in expression of extracellular matrix factor genes in 24

SYMBOL	GENE DESCRIPTION	FOLD
	**EXTRACELLULAR MATRIX**	
***Olfm4***	olfactomedin 4, also OlfD, hOlfD, ECM glycoprotein that facilitates cell adhesion.	480
***Mgp***	matrix Gla protein	5.1
***Col21a1***	collagen, type XXI, alpha 1	4.8
***Novel***	similar to AMY cluster collagen COL25A1	4.3
***Col24a1***	collagen, type XXIV, alpha 1	3.5
***Lox***	lysyl oxidase	3.4
***Ecm1***	extracellular matrix protein 1	3.2
***Pxdn***	peroxidasin homolog (Drosophila)	2.9
***Col7a1***	collagen, type VII, alpha 1	2.9
***Sod3***	superoxide dismutase 3, extracellular	2.6
***Srrm4***	serine/arginine repetitive matrix 4	2.4
***Fbn3***	fibrillin 3	2.3
***Col27a1***	collagen, type XXVII, alpha 1	2.0
***Col15a1***	collagen, type XV, alpha 1	−2.0
***Dag1***	dystroglycan 1 (dystrophin-associated glycoprotein 1)	−2.0
***Emilin2***	elastin microfibril interfacer 2	−2.1
***Nfasc***	neurofascin, L1 family immunoglobulin cell adhesion molecule with multiple IGcam and fibronectin domains: neurite outgrowth, neurite fasciculation	−2.2
***Nid2***	nidogen 2 (osteonidogen) binds collagens I and IV and laminin	−2.2
	**FIBRONECTIN**	
***Elfn2***	extracellular leucine-rich repeat and fibronectin type III domain containing 2	−2.9
	**METALLOPEPTIDASES**	
***Adam11***	ADAM metallopeptidase domain 11	3.0
***Adamts10***	ADAM metallopeptidase with thrombospondin type 1 motif, 10	2.5
***Ermp1***	endoplasmic reticulum metallopeptidase 1	−2.0
***Adam17***	ADAM metallopeptidase domain 17	−2.1
***Cndp1***	carnosine dipeptidase 1 (metallopeptidase M20 family)	−2.2
	**METALLOPEPTIDASE INHIBITOR**	
***Timp1***	TIMP metallopeptidase inhibitor 1	4.4
	**METALLOTHIONINES**	
***Mmp1***	matrix metallopeptidase 1 (interstitial collagenase)	104
***Mmp15***	matrix metallopeptidase 15 (membrane-inserted)	−2.4
	**PROTEOGLYCANS**	
***Papln***	papilin, proteoglycan-like sulfated glycoprotein	152
***Bcan***	brevican	2.1
***Novel***	similar to HS2ST1 heparan sulfate 2-O-sulfotransferase 1	−2.6
***Extl1***	exostoses (multiple)-like 1	−3.0
	**LAMININS**	
***Lamc3***	lamin gamma 3	4.8
***Lamb2***	lamini beta 2	2.6
***Lamc2***	laminin gamma 2	2.4
***Lamc1***	laminin gamma 1	2.2
***Lama4***	laminin alpha 4	2.0
	**INTEGRINS**	
***Itgb1bp2***	integrin beta 1 binding protein (melusin) 2	6.2
***Itga2b***	integrin, alpha 2b (platelet glycoprotein IIb of IIb/IIIa complex, antigen CD41)	6.1
***Itga4***	integrin, alpha 4 (antigen CD49D, alpha 4 subunit of VLA-4 receptor)	−57

Change in expression of extracellular matrix (ECM) factor genes 24 h following spinal cord injury at P28, subdivided into ECM, metallopeptidases, inhibitors, proteoglycans, laminins and integrins.

**Table 4:** Change in expression of channel, synapse, neural receptor, actin, myosin and related genes 24

SYMBOL	GENE DESCRIPTION	FOLD
	**SYNAPSES, NEURAL RECEPTORS & CHANNELS**	
***Gpr26***	G protein-coupled receptor 26	6.6
***Trpc7***	transient receptor potential cation channel, subfamily C, member 7	4.6
***Hcrt***	hypocretin (orexin) neuropeptide precursor	3.8
***Novel***	Similar to UNC80 unc-80 homolog (C. elegans)	3.7
***Sytl1***	synaptotagmin-like 1	3.0
***Fchsd1***	FCH and double SH3 domains 1	3.0
***Adora2a***	adenosine A2a receptor	3.0
***Fam40b***	family with sequence similarity 40, member B, correct symbol STRIP2	3.0
***Clcn2***	chloride channel, voltage-sensitive 2	2.6
***Bzrap1***	benzodiazapine receptor (peripheral) associated protein 1	2.6
***Mast1***	microtubule associated serine/threonine kinase 1	2.4
***Trpt1***	tRNA phosphotransferase 1	2.4
***Stxbp2***	syntaxin binding protein 2	2.3
***Mcoln1***	mucolipin 1	2.3
***C10orf10***	chromosome 10 open reading frame 10	2.3
***Ano8***	anoctamin 8	2.1
***Cacnb3***	calcium channel, voltage-dependent, beta 3 subunit	2.1
***Adrbk2***	adrenergic, beta, receptor kinase 2	2.0
***Camkk1***	calcium/calmodulin-dependent protein kinase 1α, modulation of neuron survival	2.0
***Syt2***	synaptotagmin II	−1.8
***Trpc4ap***	transient receptor potential cation channel, subfamily C, member 4 assoc prot	−2.1
***Gprc5b***	G protein-coupled receptor, family C, group 5, member B	−2.1
***Ncs1***	neuronal calcium sensor 1	−2.1
***Kif1b***	kinesin family member 1B	−2.2
***Gab1***	GRB2-associated binding protein 1	−2.2
***Kcnj10***	potassium inwardly-rectifying channel, subfamily J, member 10	−2.3
***Shroom2***	shroom family member 2	−2.5
***Syn3***	synapsin III	−2.5
***Gpr17***	G protein-coupled receptor 17	−2.6
***Gabrb1***	gamma-aminobutyric acid (GABA) A receptor, beta 1	−2.9
***Gpr75***	probable G-protein coupled receptor 75	−2.9
***Kcnj12***	potassium inwardly-rectifying channel, subfamily J, member 12	−5.8
***Novel***	similar to CLCNKA/CLCNKB chloride channel	−27
	**ACTIN, MYOSIN & RELATED**	
***Trdn***	triadin	206
***Myl1***	myosin, light chain 1, alkali; skeletal, fast	139
***Mylpf***	myosin light chain, phosphorylatable, fast skeletal muscle	22
***MYH4***	myosin, heavy chain 4, skeletal muscle	18
***Acta1***	actin, alpha 1, skeletal muscle	8.6
***Mybpc3***	myosin binding protein C, cardiac	4.8
***Parvg***	parvin, gamma	4.7
***Novel***	LOC100015891 Similar to Myosin heavy chain	4.6
***Pgam2***	phosphoglycerate mutase 2 (muscle)	4.3
***Myof***	myoferlin	4.2
***Nexn***	nexilin (F actin binding protein)	4.0
***Myo19***	myosin XIX	3.7
***Tpm2***	tropomyosin 2 (beta) member of the actin filament binding protein family	3.1
***Miox***	myo-inositol oxygenase	2.9
***Speg***	SPEG complex locus	2.8
***Novel***	similar to actinin alpha, F-actin cross-linking protein	2.8
***Tagln***	transgelin 22 kDa actin-binding protein	2.7
***Ryr3***	ryanodine receptor 3 brain ryanodine receptor-calcium release channel	2.7
***Ttll9***	tubulin tyrosine ligase-like family, member 9	2.6
***Neb***	nebulin	2.6
***Myo1f***	myosin IF	2.6
***Novel***	LOC100014836 similar to ACTA1 actin, alpha 1, skeletal muscle	2.2
***Popdc3***	popeye domain containing 3	−2.2
***Kif13b***	kinesin family member 13B	−2.3

Change in expression of channel, synapse, neural receptor, actin, myosin and related genes 24 h following spinal cord injury at P28. Note that myosin and actin genes are important structural and functional components of synapses.

### Changes in gene ontology

There was a striking difference in the number of genes that changed their expression level at the two ages; many more were changed at P28 than at P7 following injury and the magnitude of many gene expression changes at P28 was much greater (Fig. 2). In addition at P7 no genes were downregulated (>-2 fold) in response to injury in contrast to many at P28. It is also striking that at P7 almost all of the genes affected were immune/inflammatory related. Only four genes identified in the P7 injury group were categorized in the other functional groups.

Twenty-four hours following injury at P28 about half as many genes were upregulated as were downregulated and the largest category upregulated was also immune/inflammatory while the largest category of downregulated genes was enzymes & metabolism. However, nearly all categories identified in the P28 injury group contained genes that were both up- and downregulated. These gene expression changes following injury at the two ages are summarized in Fig. 2.

### Gene expression in postnatal *Monodelphis* spinal cord 24 h following transection at P7

**Figure 3:**
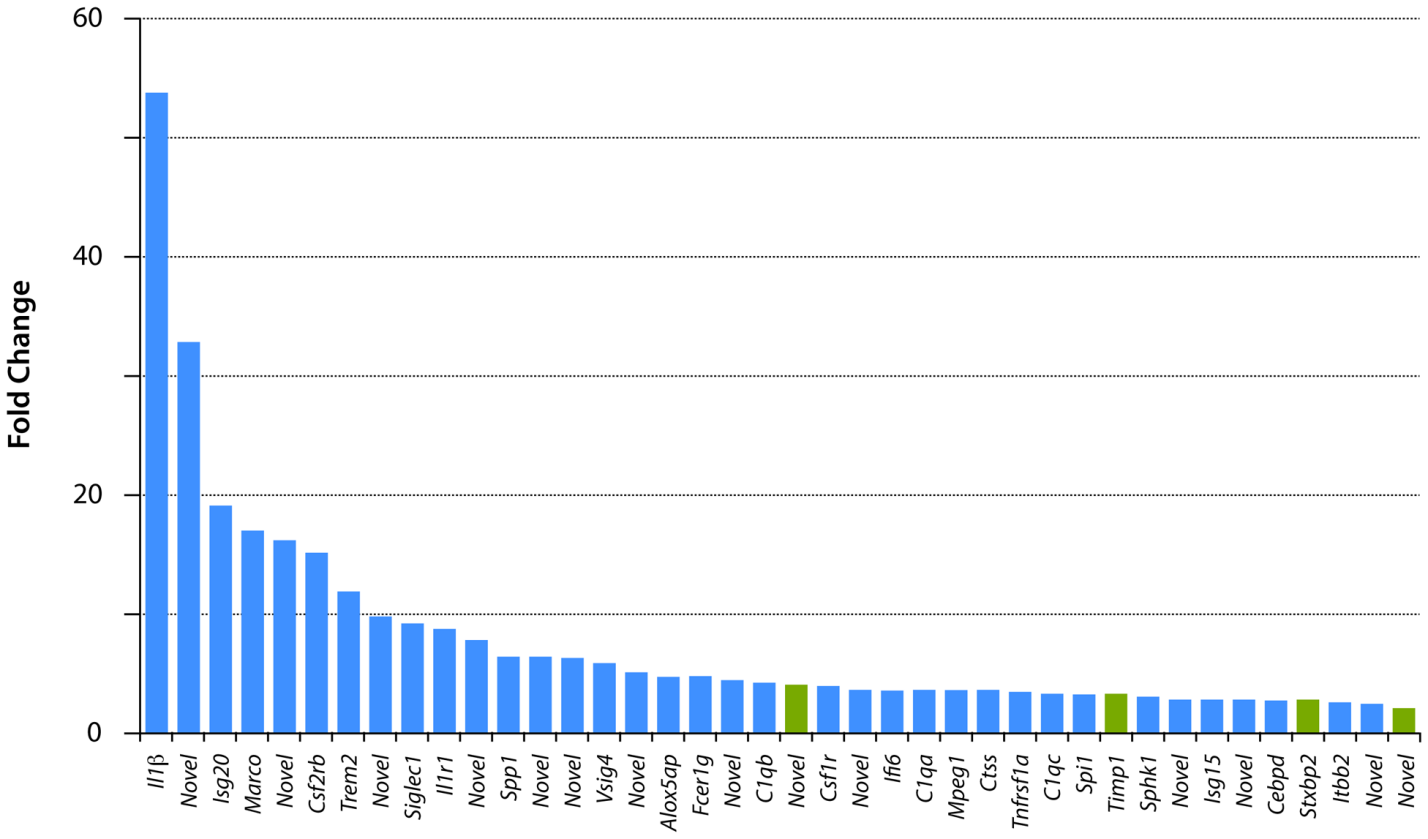
Changes in gene expression 24 Only 40 genes changed their expression levels by ≥2 fold. All were upregulated. See Table S1 for gene descriptions and statistics. There were 12 “novel” genes; search of GO categories showed that these have immune/inflammatory properties. Note that only four of the genes in this figure (green bars) are not in the immune/inflammatory category (blue bars).

At P7+24 h only 40 genes showed a significant expression change of ≥2 fold (Fig. 3 and Table S1); all of these were upregulated, none was downregulated. The gene descriptions and details of the statistical analysis are shown in Table S1 and illustrated in Fig. 3. Thirty-six of these genes were in the immune/inflammatory group (Table S1); the highest expressed of these were interleukin 1β, which was 54 fold higher than uninjured control spinal cord and a *novel* gene (C-C motif chemokine similar to *Ccl8* and *Ccl13*) which was 33 fold higher than uninjured control spinal cord. The four non immune-related genes upregulated at this age (green in Fig. 3) were the metalloproteinase inhibitor *Timp* (3.2 fold), one novel gene related to cell proliferation and apoptosis (similar to *Samd9/Samd9l* sterile alpha motif domain 9), which was upregulated 4 fold, the syntaxin binding protein *Stxbp2* was upregulated 2.7 fold and a novel gene similar to MT3 Metallothionein 3, upregulated 2 fold. It has been shown before that in adult spinal cord injury, one of the main functional groups of genes to show regulatory changes soon after injury is the inflammatory group (e.g., [16]). The finding that almost all of the gene expression changes identified at 24 h after spinal cord injury at P7 were immune/inflammatory genes with only single members of other gene families shown to have marked expression changes, suggests that the overwhelming inflammatory response at this age may reflect a general response to injury. It does itself appear to affect the outgrowth of neurites, which has previously been shown to be profuse at this age [21], [22]. The absence of an immune/inflammatory response to injury in isolated postnatal *Monodelphis* spinal cord [28], [29] supports this conclusion; this is discussed further below. Given the successful growth of axons across the lesion site at this age it would appear that the injury does little to interfere with the process, because most of the known genes associated with axon growth or inhibition were unaffected.

The immature state of the immune system at the end of the first week of postnatal life in *Monodelphis* may help to explain the complexity of immune/inflammatory genes upregulated at this time, compared with P28 (see below). That the majority of immune/inflammatory genes are upregulated is consistent with responses that are generally associated with non-specific or innate cell types, and with the early stage of development at P7/P8. At the end of the first week of postnatal life in the opossum, the αβ T cell receptor (*Tcr*) is present, although the thymus itself is still relatively immature [44]. T cells expressing the *γδTcr*, a lineage that has been implicated in playing a role in wound healing, are first detectable at P8 [44], [45]. The ontogeny of antibody producing B cells is similarly at an early transitional point. Cells committed to the B cell lineage are found in prenatal embryos and within the first 24 postnatal hours B cells that have rearranged their heavy chain antibody genes are detected [46]. But the first wave of light chain gene rearrangements necessary for developing mature B cells is not detected until P7 at the earliest. T and B cells at this stage are fairly limited in their diversity, most likely consistent with low numbers of cells. Given the immature state of the adaptive side of the immune system it may not be surprising that components of the innate immune system such as complement components *C1q* and *Factor B* are upregulated.

**Figure 4:**
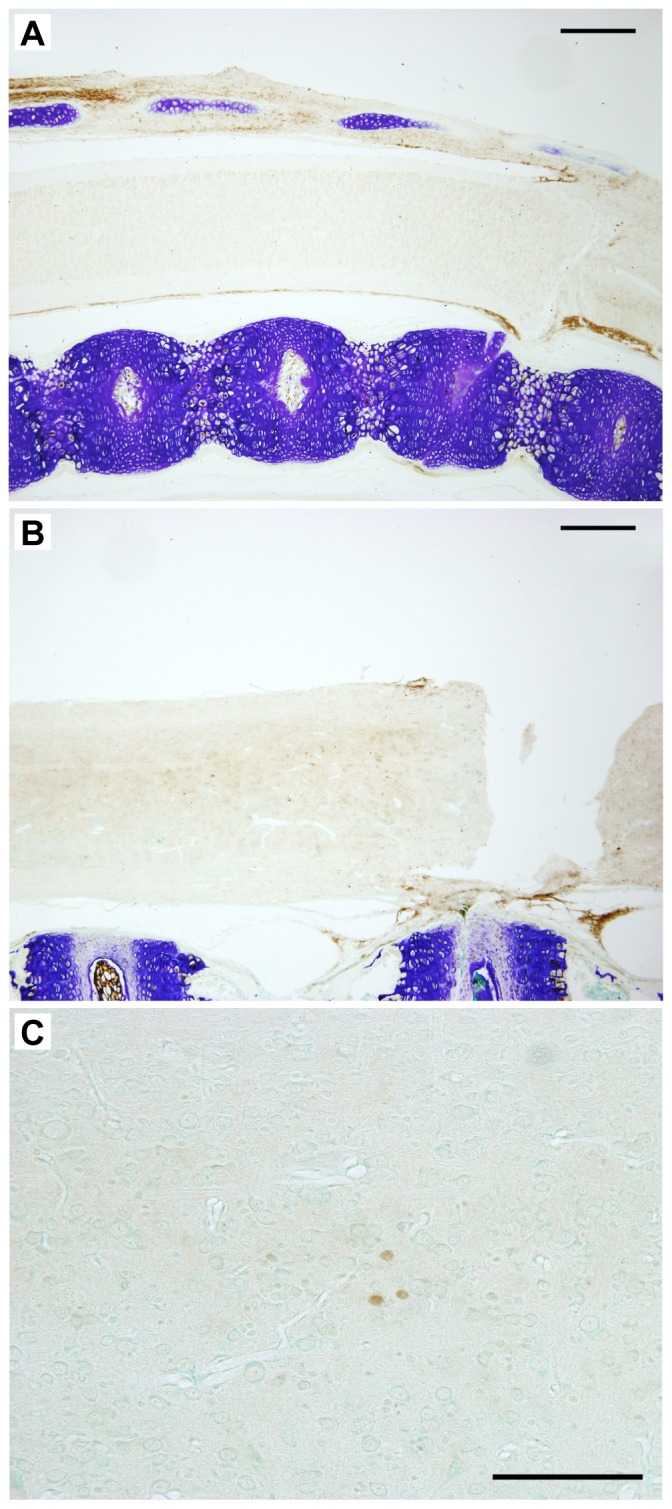
Interleukin-1β in *Monodelphis* spinal cord 24 hours after a complete transection at P7 or P28. In the segment of the cord rostral to the site of injury Il-1β was detected using cross-reacting antibodies to the human cytokine. Note strong immunopositive signal in the tissue surrounding the cords at P7-injured (A) and P28 (B) but lack of significant staining within the spinal tissue especially at P7 (A). One day following injury at P28 a few immunopositive cells with the general morphology of monocytes were detected, especially in segments of the cord more rostral to the injury (C). Scale bars A, B = 500 µm, C = 100 µm.

Upregulated transcripts such as those encoding Sialic acid-binding immunoglobulin-type lectins (*Siglec*s) and *Vsig4* have been implicated in inhibitory regulation of inflammation and adaptive immune responses through regulation of B and T cells. *Siglec*s are involved in the cell adhesion and phagocytosis, among other things, and are expressed on a variety of immune system cells including macrophages. They have been implicated in binding pathogen associated molecular patterns (*Pamp*s), transmitting inhibitory signals and participating in B cell tolerance (reviewed in [47]). Likewise, *Vsig4* is a receptor for the complement component 3 fragments, *C3b* and *iC3b* and has been implicated in being a negative regulator of T cells [48]. We were only able to obtain antibody cross-reactivity for one of the protein products of the large number of genes that showed upregulation (Il-1β). Its distribution in P7 and P28 spinal cord 24 h after injury is illustrated in Fig. 4 (for description see below).

**Figure 5:**
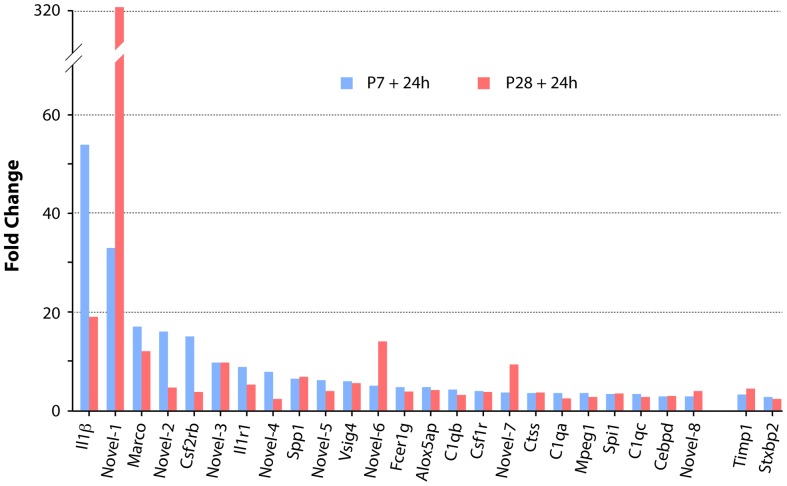
Comparison of expression levels of the twenty-six genes that changed expression in spinal cord 24 h following transection at both P7 and P28. All but two of these genes (Timp1 and Stxbp2) were in the immune/inflammatory category. The magnitude of the expression changes was generally similar at the two ages, but more genes (six: IL1β, MARCO, novel-2, CSF2RB, IL1-R1, novel-4) showed greater upregulation at P7 than at P28 (three: novel-1, novel-6, novel-7). A search of Ensembl, NCBI “Gene” and GO categories showed that all of these novel genes are involved in immune/inflammatory functions (see Tables S1 and S2).

Only two genes at P7 that might be expected to be related to a neural response to injury were upregulated >2 fold (Table S1). These were metallopepetidase inhibitor 1 (*Timp1*, 3.2 fold) and syntaxin binding protein 2 (*Sxbp2*, 2.7 fold). As these were also similarly upregulated following injury at P28 (Fig. 5) it seems unlikely that they account for successful neurite outgrowth at P7, which was lacking at P28.

### Gene expression in postnatal *Monodelphis* spinal cord 24 h following transection at P28, including comparison with injury at P7

Examination of the whole gene list shows that at P28+24 h there were 332 genes that increased their expression by 2 fold or more (Table S2); 149 genes reduced their expression by 2 fold or more. This contrasts with the response at P7 when no genes were found to be downregulated (Fig. 2 and Fig. 3, Table S1). For comparison of the response at 24 h after injury, all 40 genes that showed a change in expression at P7+24 h (Table S1) are compared with the top 40 genes that increased in expression at P28+24 h (from Table S2 as shown in Fig. 5). It is noteworthy that several genes upregulated following injury at both ages showed a much greater change at P28+24 h than even the top gene at P7+24 h (Fig. 5 and Cf Tables S1 and S2). Genes that changed expression 24 h following spinal transection at P28 are listed by functional category in Table S3 and are next considered.

#### Immune-inflammatory genes

Seventy-four genes were classified as immune-inflammatory at P28 (Fig. 2 and Table S3); of these 66 were upregulated and the remaining 8 were downregulated. Twenty-three of the immune/inflammatory genes upregulated following injury at P28 were also upregulated following injury at P7 (Fig. 5) however, only 2 were upregulated substantially more than following injury at P7 (a novel C-C motif chemokine similar to *Ccl8* and *Ccl13*, 322 compared to 33.4 fold and C type lectin/mannose receptor similar to *Mrc1*, 9.3 compared to 3.6 fold); the others were either upregulated to a similar extent or only marginally more than at P7 (Fig. 5).

The difference in the response between P7 and P28 is in the complexity. The much greater P28 response in immune/inflammatory genes may be a reflection of the state of development of the immune system. By P28 the opossum immune system is fully mature in cellular composition [46], [49]. There were 44 additional immune/inflammatory genes upregulated after injury at P28 and 9 that were downregulated (Table S3); none of these showed detectable expression changes at P7. The distribution of Il-1β immunostaining in P28 spinal cord 24 h after injury is illustrated in Fig. 4. In P7 injured cords no immunoreactivity for the cytokine could be detected in the cord but was clearly visible in the connective tissue and forming bone surrounding the spinal cord, confirming cross-species cross-reactivity (Fig. 4A). In contrast in P28 injured cords Il-1β immunoreactivity was detected in several cells of monocytic appearance mostly in close proximity to the central canal (Fig. 4B&C). These cells were present in spinal segments both rostral (Fig. 4B&C) and caudal to the site of the injury. They were not visible in control cords nor were they present in P7 injured tissue.

#### Blood-related

At P28, 24 h after injury 25 genes in this functional group were upregulated between 2 fold (von Willebrand factor precursor) and 99 fold (novel, haptoglobin-related protein; see Table S5). Only 2 genes in this category were upregulated following injury at P7: a *novel* gene (Similar to Gp6 glycoprotein VI (platelet)) was upregulated 7.8 fold compared to 2.3 fold at P28 and *Trem3* (triggering receptor expressed on myeloid cells 2) which was upregulated 12.4 fold at P7 but was unchanged at P28. One gene in this group was downregulated at P28: *Thbs2* (-4 fold). The observation that bleeding following operation on the cords at P28 was greater than at P7 (Fig. 1) perhaps accounts for the presence of some of the much greater number of genes with changed regulation at P28 (see Fig. 2).

#### Neurite inhibitory, growth/guidance and extracellular matrix genes

Studies of spinal cord injury in adult animals have shown upregulation of genes with protein products that inhibit neurite outgrowth following axonal injury by a variety of mechanisms. Many of these have been targets for therapies aimed at improving function following spinal cord injury. The main groups are:

- Those associated with myelin and the ephrin genes (e.g., [20], [50]–[52]).
- Extracellular matrix genes, including metallopeptidases and proteoglycans [20].
- RhoA activation by myelin associated inhibitors and chondroitin sulphate proteoglycans [43].
- Genes involved in axon growth and guidance [53].

Only *Timp1* (tissue inhibitor of metalloproteinase) in this category was found to change expression following injury at P7 (3.2 fold, Table S1). Genes that increased or decreased their expression in this category at P28 are listed in Table 2.

**Figure 6:**
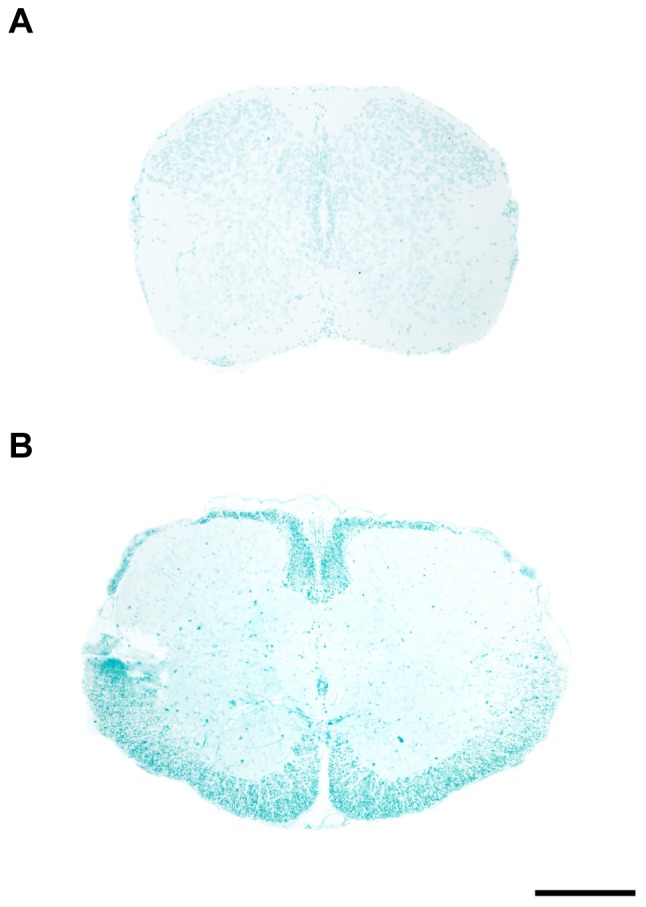
Myelin staining in the developing spinal cord of *Monodelphis domestica*. Transverse sections through thoracic spinal cord of P8 (A) and P29 (B) spinal cord stained with Luxol fast blue (LFB). There was no LFB stained myelin at P8 (A) but relatively well- developed myelin was present at P28 (B). However even at P28 the myelination is only beginning to appear (first detected between P21 and P28) and does not reach adult levels until several weeks later (not illustrated). Dorsal is uppermost. Scale bar is 500 µm.

Many inhibitory factors converge on Rho, an intracellular GTPase. These include myelin-derived inhibitory factors, semaphorins, chondroitin sulphate proteoglycans (CPSGs), ephrins, netrins and repulsive guidance molecules (RGMs; see [43]). Neither *Rho* nor its downstream effector Rho kinase (*Rock*) changed expression by 24 h after spinal cord injury at P28. Four Rho family members were identified that change expression level after injury at P28. Amongst other Rho family members, two were upregulated 3 fold (*Arhgap6*, *Gmip*) and 2 were downregulated 2 fold (*Arhgef10, Arhgap35*) see Table 2. Thus even if any of the numerous upstream neurite inhibitory factors changed expression after injury, it seems unlikely they would be effective in influencing neurite outgrowth. In fact, very few were found to change expression. Following injury at P28, none of the seven myelin-associated genes identified was upregulated. Five did not change expression (*Rtn4* also known as *Nogo*, *Mpz, Mag, Mog, Ngr*) and only two were marginally downregulated (*Mbp, Plp1*). These findings are consistent with the morphological observation that myelination is not complete at this stage of spinal cord development (Fig. 6).

Luxol Fast Blue stain for myelin demonstrated that the process of myelination in the thoracic region of *Monodelphis* spinal cord does not begin until about 3 weeks of age. There was no myelin staining at P8 (Fig. 6A). By P28 the distinctive myelin staining was apparent; however, the thickness of the myelinated white matter was still smaller than in the adult (compare Fig. 6B with Fig. 6A in [25]). From preliminary studies we have established that myelination is relatively rapid after P28 and by P35 the staining pattern of the thoracic cord is very similar to that in the adult (unpublished observations).

Of seven ephrin and nine ephrin receptor genes identified, only Ephrin-A4 (*Eph4a*) was upregulated (5 fold). *Eph4A* -/- mutant mice have been suggested to show greater axon growth after spinal cord injury [54] but others have not confirmed this [55]. Even if *Eph4A* is involved in inhibition of axon growth the lack of change in *Rho/Rock* expression suggests it would not have contributed in the present experiments. Equally, myelin inhibition is unlikely to have contributed to the failure of axons to grow across a lesion made at P28.

Twenty-one genes associated with axon growth and guidance during spinal cord development were identified as having changed expression levels following injury at P28 (Table 2). Eight were downregulated 2 to 3 fold and could therefore have possibly contributed to the lack of axon growth. The others were upregulated (Table 2) including *Hhipl2* (hedgehog interacting protein-like- 2, 6.5 fold) and *Sgsm3* (small G protein signalling modulator 3) and are thus unlikely to have contributed to lack of axon growth at P28. Amongst six netrin genes identified, only *Ntn5* was marginally upregulated (2.1 fold) and amongst seven CSPGs only one was marginally upregulated (Brevican, *Bcan*, 2 fold). Neither of the repulsive guidance molecules, *Rgma, Rgmb*, changed expression following injury.

Thirty-nine genes with extracellular matrix products, including fibronectin, metallopeptidases, metallothionines, laminins, integrins and proteoglycans showed changed expression following injury at P28 (Table 3). Of these, twenty-six were upregulated 2 to 480 fold and thirteen were downregulated (2 to 3 fold) except for *Itga4* which showed expression levels 57 fold less than controls). The most strikingly upregulated were olfactomedin 4 (*Olfm4*, 480 fold), papilin, proteoglycan-like sulfated glycoprotein (*Papln* 152 fold, see below) and matrix metallopepetidase 1 (*Mmp1*, 104 fold). *Olfm4* is a member of the 4-member olfactomedin family. Originally identified as the human granulocyte colony-stimulating factor stimulated clone-1 (hGC-1; [56]), *Olfm4* has been suggested to be specifically expressed in gut and pancreas [57] in contrast to *Olfm1*, which was reported to be specifically expressed in brain [58]. It is an extracellular matrix glycoprotein that facilitates cell adhesion by binding to cadherin and lectins [57], although it is not clear if this is its function in the spinal cord. *Olfm4* has been implicated in suppressing inflammation. Mice deficient in *Olfm4* have a stronger anti-bacterial response including enhanced inflammation to *Helicobacter pylori* [59]. However, it may be that its function in the extracellular matrix of the spinal cord may be more relevant to the present study.

Using western blotting and immunocytochemistry MMP1 has been found to be increased 24 h following spinal cord injury in rats, [60] and in human postmortem studies using immunocytochemistry [61]. Its gene expression does not appear to have been studied. The protein has been localized in macrophages and astrocytes in the human study [61] but also in neurons in the rat study [60]. MMPs are a family of peptidases that degrade extracellular matrix proteins. Early increased expression of for example *Mmp-9* is thought to have deleterious effects whereas the later upregulation of MMP-2 may be beneficial as part of the repair process [62]. The role of MMP-1 following spinal cord injury is unknown but given its early and substantial upregulation in P28 *Monodelphis* spinal cord following injury described here, it may be contributing to the lack of neurite outgrowth at this age.

Proteoglycans, particularly chondroitin sulphate proteoglycans (CSPGs) are known to be upregulated following injury to adult spinal cord and are thought to be a major component of neurite inhibition following injury [20]. These proteoglycans were not found to be upregulated in the current study (see above). However, a novel gene similar to papilin, *Papln* proteoglycan-like sulfated glycoprotein was upregulated 152 fold; this gene has not previously been identified in spinal cord but perhaps it also has neurite inhibitory properties as in the case of CSPGs. Several collagens have been identified in the post injury scar that restricts neurite outgrowth following spinal cord injury in the adult [20]. In the present study three collagen genes were upregulated (Table 3) and may have contributed to the lack of neurite outgrowth at P28, as collagen is an important component of the glial scar [20]; one was marginally downregulated (-2 fold). It seems unlikely that the rather modest changes in expression of metalloproteinase genes, some of which increased and others decreased (Table 3), would have contributed to the lack of neurite outgrowth at P28. Of 13 laminins, 5 were upregulated 2.0–4.8 fold (Table 3).

Integrins are an important receptor family promoting axon growth during development and regeneration following injury to peripheral nerves. They interact with the extracellular matrix factors described above (e.g. collagen, laminin, tenascin, vitronectin). Integrins are cell surface proteins. They bind to molecules in the extracellular matrix and transduce extrinsic cues that regulate the cytoskeleton leading to modulation of axon growth (for references see [63]). In contrast to their activity in developing nervous system and peripheral nerve regeneration, there appears to be little change in integrin expression following injury to the adult central nervous system [63]. Integrins are also present on the surface of leukocytes; for example, antibodies to integrin α4νβ1 reduced neutrophil and monocyte/macrophage influx into adult spinal cord following injury [64]. In the present study there was a substantial reduction (57 fold) in expression of *Itga4* (integrin α4) following injury at P28. Lane et al. [24] reported a small infiltration of granulocytes at 24 h following spinal cord transection in *Monodelphis* at P7. At P14 the infiltration was 2–3 times larger but was delayed compared to P7 injuries. This may explain why we do not see many granulocytes 24 hours after injury in the P28 spinal cords. However, we observe some monocytic cells as illustrated in Fig. 4. Only two other integrins (*Itgibp2, Itga2b*) showed a change in expression, increasing about 6 fold in both cases. However, these integrins have not previously been identified in spinal cord so their possible role in lack of neurite outgrowth is unclear.

Rho signaling proteins regulate the dynamics of cytoskeleton and cell motility [65]. Rho appears to be the common target of the main mechanisms that limit neurite outgrowth following spinal cord injury: myelin inhibitors, CSPGs, and guidance inhibitors [43]. Given the rather small and opposing changes in expression of Rho family genes and of many of the genes that target Rho following injury in adult spinal cords (see above) it seems unlikely that Rhos and inhibitory factors that target them contribute to the lack of axon growth following injury at P28. However, many of the axons passing through the site of injury have their cell bodies of origin in the brainstem, where changes in Rho genes might be expected to be manifest. Changes in brainstem gene expression will be the subject of a separate report (Saunders *et al.,* in preparation) but preliminary examination of the RNA-Seq data suggests that here too changes in expression of Rho-related genes following spinal cord injury are also marginal.

#### Cell adhesion and intercellular junctions

There were twelve genes in this functional category (S3). Only four were upregulated (2.2. to 3 fold). The other seven decreased their expression (-2 to -3 fold). Most of these genes have not previously been identified in spinal cord. *Pdch1* appears to play a role in spinal cord development in the mouse [66] and *Pdch18* in chick and zebrafish developing spinal cord [67], [68]. *Gjc2* encodes the gap junction protein connexin-47; a mutation of this gene has been associated with hypomyelination [69]. Of all the genes identified in this group only *Icam-1* has been found to change expression in injured adult spinal cord. Thus its expression has been found to increase following spinal cord injury in rats; endothelial cell upregulation of *Icam1* was suggested to increase adhesion and extravasation of leukocytes 1–2 days following injury [70]. The possible importance of ICAM-1 in the response to injury is indicated by the report that intravenous injection of ICAM-1 monoclonal antibody 30 min after spinal cord injury in rats reduced motor disturbance and enhanced recovery [71].

#### Channels, synapses and neural receptors

In previous studies of adult spinal cord it has been reported that some ion channel genes, including those for sodium, potassium and calcium are downregulated shortly after injury [16]. In the present study four channel genes were downregulated (Table 4), including one potassium channel (*Kcnj12*, -5.8 fold) and one chloride channel (a novel gene, similar to *Clcnka/Clcnkb* chloride channel) that was markedly downregulated (27 fold). In addition three channel genes were upregulated (*Trpc7, Clcn2, Cacnb3*, Table 4).

In reviewing a large number of microarray studies of adult rodent spinal cord Verhaagen *et al.* [16] also reported genes encoding enzymes involved in neurotransmitter synthesis and genes encoding proteins involved in synaptic vesicle transport and docking as well as expression of glutamate receptors, which were generally downregulated following spinal cord injury. In our dataset in this category, there were five genes that were upregulated (2 to 3 fold) and six that were downregulated (2.1 to 2.9 fold) see Table 4. Myosin and actin are important for normal structure and function of neural synapses [72]–[74]. Genes related to these protein products have been listed separately in Table 4. Twenty-three were identified with significant changes in expression; all but one (*Popdc*, -2.2 fold) was upregulated. Of those upregulated, some increased their expression very substantially: *Triadin* (206 fold), *Myl1* (139 fold), *Mylpf* (22 fold) and a *novel* gene *LOC100027326*, myosin-4-like (18 fold). Triadin was first discovered as an important intrinsic membrane glycoprotein in the sarcoplasmic reticulum of skeletal muscle [75]. Although initially thought to be exclusive to skeletal muscle, according to Dulhunty *et al*., [76] it has been found in a range of tissues. However, it does not seem to have been identified previously in spinal cord, but there is one report of Triadin 2 (the cardiac isoform) in mouse brain [77] together with the intracellular calcium channel proteins ryanodine receptors (RyR) 1 and 3. *Ryr3* was upregulated in the present study (Table 4). Ryanodine receptors and Triadin are essential components of sarcoplasmic Ca^2+^ transduction in skeletal muscle [76]. Their presence in spinal cord suggests that they may have a role in Ca^2+^ storage and release there too. *Myl1* and the *novel* gene (*myosin-4-like*) have not been identified in spinal cord previously. *Mylpf* a gene involved in muscle contraction has been found to be downregulated in the spinal cord in response to acrylamide [78]. These genes and those listed in Table 4 show changes in expression following injury that is presumably related to changes in synaptic function at later stages after the injury. They may reflect some of the changes in gene expression and neural circuits in opossum spinal cord caudal to the injury site following injury at P28 [26]. These may be important for the weight bearing locomotion displayed by these animals when adult, in spite of the lack of axonal growth across the lesion (25). However, it seems unlikely that these changes in synapse-related genes are contributing to this lack of axon growth following injury at P28.

#### Apoptosis and ubiquitin

Only one apoptosis-related gene was upregulated at P7 following injury: a *novel* gene, related to *Samd9*. In contrast, five genes with products that are involved in apoptosis were upregulated 3 to 6 fold in the P28 injured cords: *Dnajb13* (*Hsp40* homologue), three *novel* genes and *Plac8* (Table S3). *Faim2*, a gene for Fas apoptotic inhibitory molecule was downregulated (−2.3 fold) as was the apoptosis-associated tyrosine kinase *Aatk* (Table S3). This suggests a degree of apoptotic activity that was not present following injury at P7. Lane *et al.* [24] reported a large increase in pyknotic cells 3 h after spinal cord transection in P7 and P14 *Monodelphis*. This returned to control levels by 24 h, which may account for the limited expression changes in apoptotic-related genes at P7 and P28 in the present study conducted 24 hours after injury (Tables S1, S2 and S3).

Four ubiquitin-related genes increased expression (4.0 to 7.6 fold, Table S3). Only one was downregulated (a novel gene similar to *Ubr3*, -2 fold). These genes, *Wsb1, Cul7, Cul9* and *Ubr3* are all genes for different E3 ubiquitin ligases that ubiquitinate various target proteins, signaling them for protein degradation. WSB1 is a hedgehog inducible ubiquitin ligase socs-box-containing WD-40 protein. Currently, there is no evidence for this process following spinal cord injury; however, it is an E3 ubiquitin ligase for thyroid hormone-activating type 2 iodothyronine deiodinase [79], which is a HIPK2-interacting protein [80]. The cullin family of genes codes for *cullin E3 ligase* scaffold protein, which interacts with *Rbx1*. In this study, we identified two cullin family members with >2 fold change, *Cul7* and *Cul9*. CUL7 has only been shown to bind with FBXW8 [81]. However, there is some evidence that this protein heterodimerizes with CUL1, facilitating polyubiquitination of target proteins [81]. The specific function of CUL7 in the spinal cord is otherwise unknown; however, CUL1 [82] and CUL3 [83] have been shown to be important during embryonic development through interaction with cyclin E. *Cul7* knockout-mice also show neonatal lethality [84]. CUL9, previously known as PARC on the other hand has been shown to bind and promote p53-dependent apoptosis [85]. The last of the E3 protein ligase genes identified is *Ubr3*, ubiquitin protein ligase E3 component n-recognin 3. This gene codes for a ligase that specifically targets the N-terminal residues for degradation [86]. Although its role has not been previously described in spinal cord injury, this ligase has been shown to play a role in olfactory and tactile sensory systems [86].

#### Vascular/angiogenesis and transporters

Three genes with vascular/angiogenic properties were upregulated 2.5 to 3.8 fold (Table S3). Of fifteen transporter genes seven were upregulated (3 to 12 fold) and eight were downregulated (−2 to −5 fold). Only two have previously been identified in spinal cord (*Sclc18a3*, vesicular acetylcholine transporter and *Atp2a1*, both downregulated). There was only one efflux transporter gene (*Abcc5*, *Mrp5*, upregulated 4.5 fold). Most of the influx transporters were ion carriers, some upregulated, others downregulated. Of two monocarboxylic transporters one (*Slc16a4*) was upregulated (8 fold) and one (*Slc16a1*) downregulated (−2 fold). The highest change in expression was that of the vitamin C transporter *Slc23a3* (12 fold). It seems unlikely that any of these changes are directly related to the lack of axon growth following injury at P28.

#### Development and structure

Thirty-one genes were classified as development and structure related genes (Table S3). Of these, twenty were upregulated, 2.1 to 21 fold and eleven were downregulated, (−2 to −11 fold). Several of these genes have important roles in neural development. For example *Fuz*, which was upregulated 21 fold, is a planar polarity gene, mutations of which are associated with neural tube defects [87]. *Hoxb1* (12 fold) and *Hoxb3* (2.4 fold) are transcription factors involved in patterning of the caudal central nervous system [88], [89]. *Hoxd4* (2.4 fold) does not appear to have been identified previously in spinal cord but *Hoxd10* has been implicated in determining motor neuron numbers and spinal nerve trajectories in lumbar spinal cord of mice [90]. There were two insulin growth factor-like genes, one upregulated (*Igf2bp2*, 2.5 fold) and one downregulated (*Igfbpl1*, −11 fold); there was also one IGF-like receptor (*Igflr1*, 5.2 fold). None of these have previously been identified in spinal cord. Only five of the remaining genes have been identified previously in spinal cord (*Tube1, Notch3, Rhou, Sort1, Vim*). Of these, tubulin protein has been reported in spinal cord injury, where it was upregulated 2.7 fold [91] compared with a 4.5 fold in gene expression, and vimentin (*Vim*) was upregulated [92]. Given that many of these genes are of unclear function in normal or injured spinal cord it cannot be concluded whether they contribute to the lack of neurite outgrowth following injury at P28, although this seems unlikely.

### Proteomic analysis in postnatal *Monodelphis* spinal cord following transection

The changes in proteomic expression have been examined in the segment of the cord rostral to the lesion compared to age-matched controls. Proteins identified from gel bands that showed changes in the density were classified under the same functional categories as those used in the RNA-Seq analysis (see above).

### Protein expression in postnatal *Monodelphis* spinal cord 24 h following transection at P7

**Figure 7:**
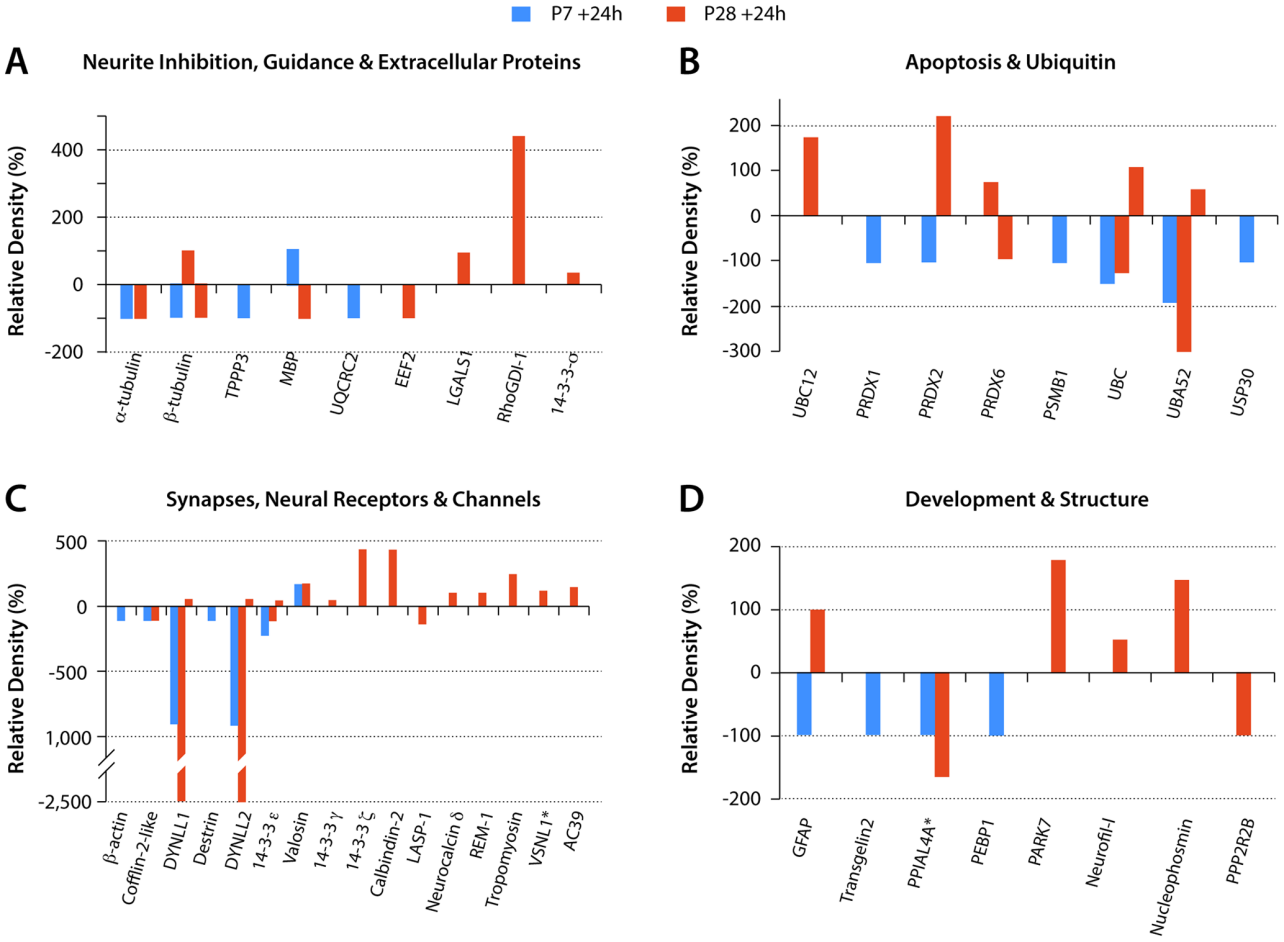
Proteins by functional groups with changed expression levels after spinal transection at P7 or P28. Estimates of protein expression levels from densitometry measurements. Values are expressed as % change from control values (100%). y axis: Relative Density (%). Proteins grouped by functions as listed in Tables S4 and S5. Abbreviations: TPPP3-Brain specific protein; UQCRC2-Ubiquinol-cytochrome c reductase core protein II; EEF2-Elongation factor 2 isoform 1; LGALS1-galactin1; MBP–Myelin basic protein; PRDX1-peroxiredoxin1; PRDX2–peroxiredoxin 2; PRDX6–peroxiredoxin 6; PSMB1-Proteasome subunit β type 1; UBC–Ubiquitin C; UBA52-Ubiquitin A-52; USP30-Ubiquitin specific peptidase 30 phosphoglycerate kinase; BC12-NEDD8-conjugating enzyme UBC12; DYNLL1- Cytoplasmic dynein light chain 1; DYNLL2-Dynein light chain LC8 type 2; VSLN1-Visinin like-1; AC39-AC39/physophilin; GFAP–glial fibrillary acidic protein; TAGLN2-transgelin 2; PPIAL4A - Peptidylprolyl isomerase A-like PEBP1 - Phosphatidylethanolamine-binding protein 1; PARK7 - Parkinson protein 7; Neurofil-L – Neurofilament-L subunit; PPP2R2B - Protein phosphatase 2 regulatory subunit B. Two proteins, VSNL1 and PPIAL4A (marked with *) are mean values as their expression levels changes were detected in more than one fraction (see Table S4 and S5 for individual changes). Note also that a few of the proteins listed showed both up- and downregulation but only in P28 injury group.

Compared to P8 age-matched controls, in cords injured at P7 and analysed 24 h later there were only four proteins with increased band intensity (upregulated). Almost all of the rest of the proteins identified at this age (twenty-six) showed reduced band intensity (downregulated); one protein was identified with both an increased and a decreased density that depended on the fraction in which the protein was separated (Tables S4 and S6). The identity of these proteins and their classification into functional categories are shown in Table S4. Four of the functional groups: Neurite Inhibition, Guidance & Extracellular; Apoptosis & Ubiquitin; Synapses, Neural Receptors & Channels and Development & Structure are illustrated in Fig. 7.

#### Neurite inhibition, guidance and extracellular proteins

One neurite inhibitory protein, myelin basic protein, was upregulated, whereas a protein that interacts with the myelin inhibitory protein, NOGO (Ubiquinol-cytochrome c reductase core protein II, see [93]) was downregulated. Three tubulin proteins were also downregulated (Fig. 7A and Table S4).

#### Apoptosis and ubiquitin-related

All six of the proteins in this group were downregulated. Three were ubiquitin-related, two were peroxiredoxins with an antioxidant protective role [94] and one was an immunoproteasome protein, which also is involved in processing of class 1 MHC peptides (http://www.ncbi.nlm.nih.gov/gene/5689). This is illustrated in Fig. 7B (data in Table S4).

#### Synapses, neural receptors and channels

Six of the seven proteins in this group were down regulated. Their functions appear to relate to intracellular structure, motility and transport. The one protein that was upregulated was a vesicle transport and fusion protein (valosin).

All of the proteins assigned to the development and structure and the metabolic-associated protein groups were downregulated; two stress response genes changed in opposite directions (Fig. 7C and Table S4).

### Development and structure

Four proteins in this group (glial fibrillary acidic protein, transgelin-2, peptidylprolyl isomerase A-like and phosphatidylethanolamine-binding protein 1) were downregulated. None was upregulated (Fig. 7D and Table S4).

#### Blood-related proteins

Two hemoglobin proteins, hemoglobin-α and hemoglobin subunit β-M showed changes in band density; hemoglobin-α was increased in Off gel fraction 12 and reduced in fraction 9 (Table S4), hemoglobin subunit β-M was increased. Neither of these proteins has been identified previously in spinal cord. It is possible that their presence was due to blood contamination in the injured cords (see Fig. 1).

### Protein expression in postnatal *Monodelphis* spinal cord 24 h following transection at P28

**Figure 8:**
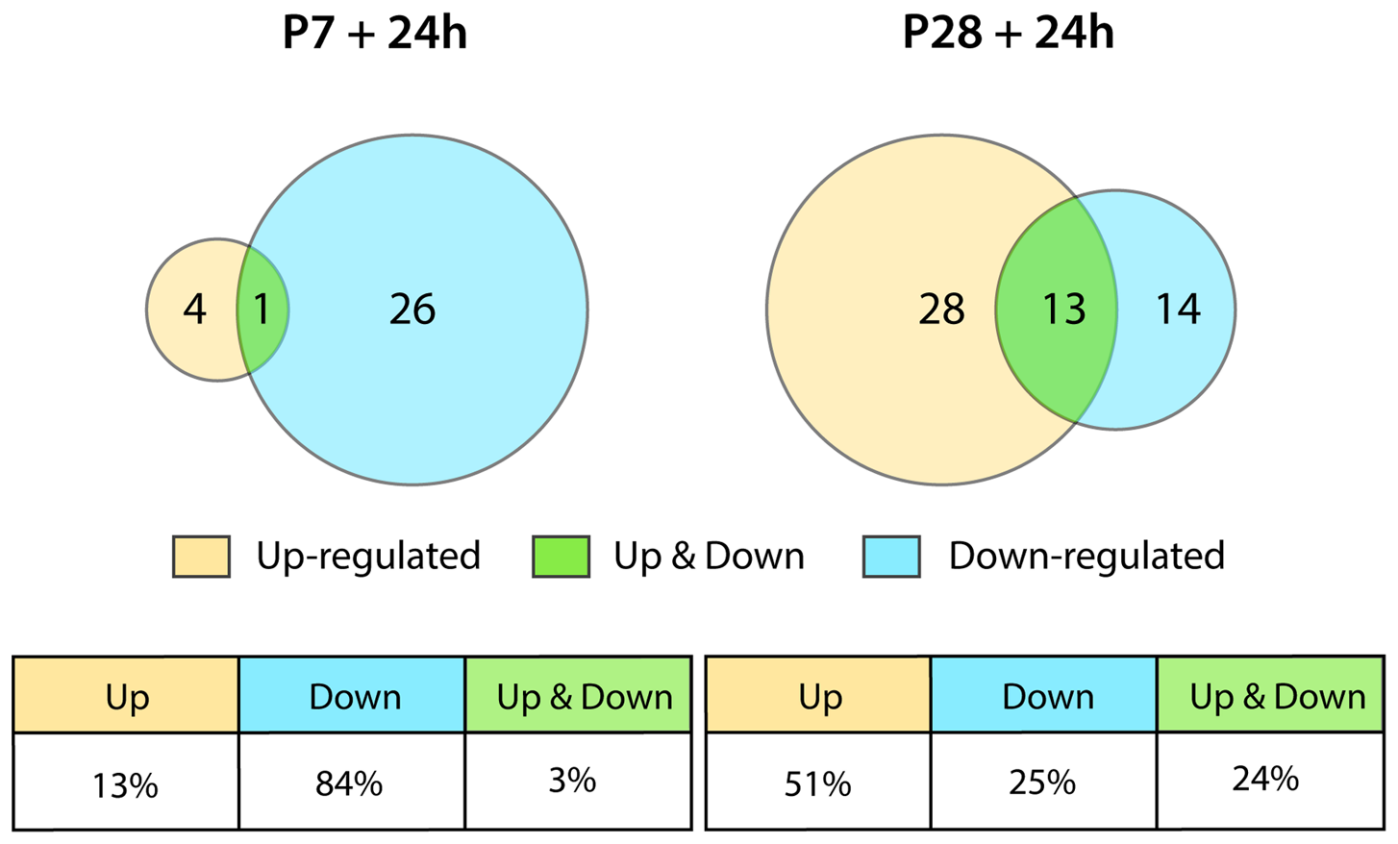
Summary of numbers of proteins that changed expression levels 24 All but five of the proteins identified at P7 as showing a change in expression after injury were downregulated. At P28 half of the proteins were upregulated.

Following injury at P28 (P28+24 h) compared to P29 controls twenty eight protein bands were identified with increased intensity, fourteen protein bands with reduced intensity and thirteen that showed both increased or decreased intensity depending on the Off-gel fraction in which they were present (Fig. 8, Table S6) most likely reflecting changes in post-translational modifications [31]. The variability in the proteome following injury at P28 is in contrast to the response to injury at P7 when almost all of the proteins identified were downregulated (Fig. 7).

#### Immune-inflammatory proteins

In contrast to the very large number of genes in this functional category that changed their regulation at P28 following injury, only three proteins were identified here (Table S5). One was *Anxa2* (annexin a2), which showed decreased expression, although the expression of its gene was unchanged. Annexin2 is involved in recruitment and activation of immune cells [95]. A protein that is likely to have immune related functions was also identified (immunoglobulin lambda-like polypeptide 5-like) and found to be upregulated (Table S5). However, its actual function appears to be unknown. A third protein was Melanocortin 1 Receptor, which is said to have anti-inflammatory and immunomodulatory effects [96]. The discrepancy between the large number of identified genes and small number of proteins is most likely to be due to the sensitivity of detection methods: proteomic analysis is not likely to detect molecules within relatively low concentration range as is the case for most cytokines. It is also possible that some of the genes detected by RNA-Seq. analysis were not yet translated into their protein products within the time frame used in this study (24 hours).

#### Neurite inhibition and guidance proteins

Unlike results following injury at P7, at P28 myelin basic protein was downregulated (Fig. 7A). On the other hand Rho GTPase activating protein, through which many of the neurite inhibitory factors act (see above) was increased. Two other proteins that have been suggested to influence neurite outgrowth (Elongation Factor 2 Isoform 1 [97] and Lectin, galactoside-binding, soluble 1 (Galectin 1) [98], [99]) changed in opposite directions (Fig. 7A, Table S5). Galectin-1 has also been described in neutrophils at the site of a spinal cord injury made 24 h earlier and in macrophages/microglia at 3 days post injury [100]. This increase in Galectin-1 protein was not accompanied by a change in transcript expression (Table S6).

#### Apoptosis and ubiquitin

Five of the eight proteins in this category were upregulated in contrast to P7 injured spinal cord when the proteins in this group were all downregulated (Fig. 7B). However, there were three proteins in this group, which were identified as a band in a different fraction that showed decreased density (periredoxin-6, ubiquitin c and ubiquitin A52, Fig. 7B, Table S5). Detailed studies of the apoptotic activity in *Monodelphis* spinal cord after injury have not been conducted, mostly due to the lack of cross-reactivity of commercially available antibodies commonly used as markers for this process.

#### Synapses, receptors and channels

There were twelve proteins in this category, including three members of 14-3-3 proteins (ε, γ, ξ) that were upregulated. These are involved in tyrosine 3-monooxygenase/tryptophan 5-monooxygenase activation signal transduction. Seven proteins involved in synaptic structure and function were upregulated, but only two (Cofilin and LIM & SH3 protein) were downregulated (Fig. 7C, Table S5). It seems unlikely that these proteins would play a role in the lack of neurite outgrowth following injury at P28, but they may indicate an early aspect of synaptic reorganisation in the injured spinal cord.

#### Development and structure

There were eight proteins identified as changing their expression in this category, including glial fibrillary acidic protein (GFAP). All but two (PPIAL4A, PPP2R2B) were upregulated (Fig. 7D, Table S5). However two of the upregulated proteins also appeared as bands in other fractions that indicated downregulation. These changes may be a reflection of developmental reorganization of spinal cord tissue rather than involvement in blocking neurite outgrowth at P28.

#### Stress response proteins

Five proteins with changed expression were identified in this category (Table S5). Two were upregulated (heat shock proteins HSP84b and HSP90B1), two were downregulated (glucose regulated heat shock protein 70kDa, protein 5; novel heat shock protein) and one (heat shock protein 90) was both increased or decreased depending on the fraction. Heat shock proteins are highly expressed cellular proteins in all species examined so far [101]. Stressors of various kinds generally result in an increase in the levels of these proteins e.g., HSP70 and HSP90 [101]. Several authors have reported this effect early after spinal cord injury in adult animals [102], [103]. However, downregulation of some heat shock proteins by various drug treatments has been reported in spinal cord injury (see [104] for references). It has therefore been suggested that both up- and down-regulatory changes in heat shock proteins may have neuroprotective effects, reflecting a complex role in protective responses to injury [104].

#### Blood-related proteins

As observed following P7 injury two hemoglobins showed a change in regulation. However, following injury at P28 hemoglobin α was decreased whereas at P7 different fractions showed either a decrease or/and an increase (Cf Tables S4 and S5). For hemoglobin β, the changes were the opposite; that is at P7 this protein was increased but at P28 different fractions showed opposite changes. In addition to these two hemoglobins, two other proteins showed an increase following injury at P28: the plasma carrier protein albumin and NADPH-flavin reductase, which is involved in heme metabolism. As suggested for P7 it may be that these blood-related proteins originate from blood contamination of the spinal cord as a consequence of the injury (see Fig. 1).

### Comparison of gene and protein expression

Based on the proteins identified, we searched for corresponding genes or family of genes associated with these proteins. Table S6 shows the matches of proteins and genes identified from RNA-Seq analysis that were up- or downregulated (proteins ±0.5 band density, genes ±2 fold change). A few proteins did not have IDs that corresponded to a gene in our RNA-Seq dataset. This may be due to incompleteness of the annotation of the opossum genome. Most of the genes corresponding to up- or downregulated proteins that we identified as changing their expression following injury at either P7 or P28 did not show changes in the RNA-Seq analysis. In only two cases did both gene and protein changed both at P28. (i) Hemoglobin beta M was identified in three different fractions. In two of these the protein was upregulated and in the third it was decreased. The corresponding gene is a *novel* opossum gene (LOC100019389) that was upregulated 2.2 fold. (ii) A second upregulated gene (*Blvrd*) corresponded to an upregulated protein (NADPH-flavin reductase) that is involved in heme metabolism. The protein products of these genes may perhaps be explained by blood contamination in the injured spinal cord. However, this would not explain the presence of these genes. Erythrocytes are no longer nucleated in opossums after birth and hemoglobin transcripts have been identified in cerebral endothelial cells of neonatal mice [105], which may thus explain the changes in gene expression found in the present study.

There are several reasons why there were so many more genes that showed changes in expression that was the case for the proteins. The main one is likely to be that the protein extraction method used was favorable for soluble cytoplasmic proteins, whereas many of the products of genes identified are associated with cell membranes and intracellular structures such as microtubules and actin filaments. It is also possible that the increased expression of some of the genes had not yet resulted in increased protein synthesis within the 24 h period following injury. In addition, as mentioned earlier, the sensitivity of the two methods is very different and many proteins whose genes showed changes are not present in cord tissue in concentrations high enough to be detected by silver staining of separated bands. In addition we have identified previously that some of the observed changes in the bands intensity are due to post translational modifications rather than changes in corresponding gene expression [32], [33].

## General Discussion

The most striking observation from this study is the large number of genes and proteins that change their level of expression within 24h after spinal cord injury in the neonatal opossum. The number of these changes was much greater following injury at P28 compared to P7, particularly when considering transcriptome changes. At P7 there were no genes that showed significant decrease (i.e., ≥−2 fold) in expression. In contrast most of (84%) of the proteins that changed expression at P28 were downregulated. This could in part be due to tissue loss following injury, thus reducing the amount of protein-containing tissue sampled but is also likely to be a reflection of the timing of these experiments: early tissue loss is followed by a later adaptation of the cellular response, as reflected by transcriptome changes detected.

The comparatively muted response to injury at P7 may indicate that the injury was introduced at an age when substantial spinal cord growth occurs as part of normal development and that this was little impaired by the trauma. Whereas by P28, when many of the spinal cord tracts are established, the response is more “adult-like” in the inability of the injured spinal cord to display a regenerative response [25]. By far the largest category of genes to respond to injury was the immune/inflammatory group. There were twenty-four genes that changed expression after injury at both ages, all increasing their expression. Nineteen changed to a similar extent at both ages (< two times difference between the ages). More genes in this category showed a larger increase in expression at P7 than at P28; only three novel genes at P28 showed substantially higher increases (322, 14 and 9.3 fold) in expression than at P7 (Fig. 5).

### *In vivo* compared to *in vitro* opossum spinal cord injury experiments

Several previous studies have assessed the importance of the developmental stage and the consequences of spinal cord injury on patterns of gene expression in the opossum [28]–[30]. In these investigations, changes in specific families of genes associated with axonal growth as well as inhibitory molecules were emphasized. In contrast, more extensive changes in gene expression were documented in the present study. There are several possibilities for these differences that relate to the experimental design of the studies. At a technical level the coverage of RNA Sequencing is potentially much greater than that of a microarray chip. In papers in which an isolated spinal cord preparation was used [29], [30] the tissues were bathed in a room temperature solution (24–25°C; [28]) during and after the spinal cord injury. Mild levels of hypothermia are known to be neuroprotective after brain and spinal cord injury and may alter specific secondary injury mechanisms including alterations in gene expression [106]–[109]. Thus, the hypothermic conditions that were used in the isolated opossum spinal cord preparation may have influenced some injury-induced gene expression changes compared to *in vivo* spinal cord injury preparations conducted at normal body temperature (31–34°C in the opossum [110]). In addition, because these opossum studies used an isolated spinal cord preparation, the involvement of acute systemic responses to spinal cord injury would also be expected to differ in comparison to *in vivo* injury conditions, due to the lack of blood circulation. Thus the well-described inflammatory response to spinal cord injury involving infiltration of different types of immune cells after experimental and clinical neurotrauma [111]–[113] would not occur in the absence of a circulation. These injury-modeling conditions may therefore help explain some differences in patterns of gene expression between the *in vitro* versus *in vivo* spinal cord injury studies. The relative contributions of lower temperature and absence of immune cell infiltration are hard to judge. The lower temperature of the *in vitro* preparations does not appear to have suppressed other aspects of the response to injury compared to the absence of an immune/inflammatory response. Since there was a lack of neurite growth across the lesion both at P13 *in vitro* and P28 *in vivo* it seems unlikely that the large number of additional immune/inflammatory genes that changed their expression at P28 compared to P7 *in vivo* was a major factor in this lack of neurite outgrowth. Also it is apparent from the Supplementary data in Mladinic *et al*., [29] that only a few immune genes were detected and they changed little in response to injury at either age. This would suggest that the lack of a circulation in the *in vitro* preparations might have been the main factor in the absence of much of an inflammatory response in these isolated preparations. In addition, it is clear from studies of injured adult spinal cord that changes in expression of immune and inflammatory genes and proteins are mainly of significance in relation to secondary injury that follows the primary injury with a time course that has been variously estimated to be days to weeks (see [114], [115]).

### To what extent may the observed changes in gene and protein expression account for the lack of neurite outgrowth at P28 following injury?

Twenty-nine of thirty-four genes suggested to influence neurite growth (Table 2) only changed their expression to a limited extent in the range +3 to −3 fold change, with equal numbers up- and downregulated. In addition, key inhibitory genes either did not change their expression (*Nogo, Mag, Mog*) or were downregulated (*Mbp, Plp1*), rather than upregulated. Thus it seems unlikely that the explanation for the lack of neurite outgrowth is accounted for by inhibitory activity of the protein products of these genes. There were similar numbers of opposing changes in protein expression for this functional group after injury at P28 (Fig. 7, Table S5) and myelin basic protein was downregulated as was its gene. Most extracellular matrix genes (Table 3) also showed relatively small and opposing changes in expression, but there were some notable exceptions. Three genes showed very large increases in expression: olfactomedin 4 (480 fold), *Mmp1* (104 fold) and *Papln* (*papilin*, proteoglycan-like sulfated glycoprotein, 152 fold). One gene in this category showed a marked decrease in expression: integrin α4 (−57 fold). Overall, there were some thirty-four genes that increased their expression by >10 fold following injury at P28 and only four that showed reduced expression of >10 fold. Of the upregulated genes almost half were in the immune and blood-related functional categories, which for reasons outlined above were probably not contributing to the lack of neurite outgrowth. Only three of the substantially upregulated genes (*Olfm4, Mmp-1*, *Papln*) and one markedly downregulated gene (*Itga4*) are in a functional category (see Table 5) in which there is evidence for effects on neurite outgrowth. Proteoglycans generally seem to have inhibitory effects on neurite outgrowth [20]. *Mmp-1* has been reported to be increased by one day after injury in adult spinal cord, but MMPs as a group appear to have rather complex effects following spinal cord injury, some deleterious and others beneficial [116], [117]. It is not known what the function of olfactomedin-4 may be in spinal cord injury, but it binds to cadherins and lectins [57]. Olfactomedin-1 promotes neurite outgrowth by binding to the Nogo A receptor complex (NgR1) and inhibiting the growth cone collapse induced by myelin inhibitors [118]. Several integrins (α6, α7, α9 and β1) have been implicated in promoting axon growth during development and in the response of the peripheral nervous system to injury [63]. Integrin α4β1 is expressed by leukocytes that invade spinal cord tissue following injury [64]. However, it has also been described as having a role in regenerating growth cones following injury to sensory neurons where it provides a signaling pathway for re-expression of fibronectin (for references see [119]). Thus its downregulation could be contributing to the lack of neurite outgrowth in P28 injured spinal cord, particularly if combined with inhibitory effects of MMP1 and papilin.

**Table 5:** Genes that changed most in expression (±≥10 FOLD) 24 h following spinal cord injury at P28.

SYMBOL		GENE DESCRIPTION		FOLD
**MOST UPREGULATED GENES**				
**IMMUNE & INFLAMMATORY**				
***Novel***		C-C motif chemokine similar to CCL8 and CCL13	322	
***Dok3***		docking protein 3	266	
***Novel***		C-C motif chemokine similar to CCL2, CCL7, CCL8, CCL11 and CCL13	197	
***Pstpip1***		proline-serine-threonine phosphatase interacting protein 1	167	
***Lrrc33***		leucine rich repeat containing 33 Official name NRROS	160	
***Novel***		similar to TARM1 T cell-interacting, activating receptor on myeloid cells 1	22	
***Il1b***		interleukin 1, β	20	
***Vav1***		VAV1 guanine nucleotide exchange factor	17	
***Osmr***		oncostatin M receptor	14	
***Lgals4***		lectin, galactoside-binding, soluble, 4	14	
***Amh***		anti-Mullerian hormone	12	
***Marco***		macrophage receptor with collagenous structure	12	
***Igsf22***		immunoglobulin superfamily, member 22	12	
***Novel***		LOC100020928 C-C motif chemokine similar to CCL3 and CCL4	10	
**BLOOD-RELATED**				
***Novel***		similar to HPR, haptoglobin-related protein	99	
***Novel***		similar to TARM1 T cell-interacting, activating receptor on myeloid cells 1	22	
**EXTRACELLULAR MATRIX**				
***Olfm4***		olfactomedin 4, ECM glycoprotein that facilitates cell adhesion.	480	
**METALLOTHIONINE**				
***Mmp1***		matrix metallopeptidase 1 (interstitial collagenase)	104	
**PROTEOGLYCAN**				
***Papln***		papilin, proteoglycan-like sulfated glycoprotein	152	
**ACTIN, MYOSIN & RELATED**				
***Trdn***		triadin	206	
***Myl1***		myosin, light chain 1, alkali; skeletal, fast	139	
***Mylpf***		myosin light chain, phosphorylatable, fast skeletal muscle	22	
***Myh4***		myosin, heavy chain 4, skeletal muscle	18	
**TRANSPORTERS**				
***Slc23a3***		solute carrier family 23, sodium-dependent vitamin C transporter 3	12	
**DEVELOPMENT & STRUCTURE**				
***fuz***		fuzzy homolog (Drosophila) cell polarity, ciliogenesis & cell movement.	21	
***hoxb1***		homeobox B1, transcription factor role in morphogenesis	12	
***des***		desmin	12	
**MEMBRANE**				
***Novel***		novel protein with potential transmembrane domains	254	
***Novel***		novel protein with potential transmembrane domains	241	
***Tmem106a***		transmembrane protein 106A	157	
**ENZYMES & METABOLISM**				
***Pstpip1***	proline-serine-threonine phosphatase interacting protein 1		167	
***Novel***	similar to ATP13A5 ATPase type 13A5		23	
**MISCELLANEOUS**				
***Ttc40***	tetratricopeptide repeat domain 40		89	
**ZINC FINGER**				
***Novel***	similar to ZC3H13 zinc finger CCCH-type containing 13		352	
**MOST DOWN REGULATED GENES**				
***Itga4***	integrin, α 4 (antigen CD49D, alpha 4 subunit of VLA-4 receptor)		−57	
***Novel***	similar to CLCNKA/CLCNKB chloride channel		−27	
***Igfbpl1***	insulin-like growth factor binding protein-like 1		−11	
***Gxylt2***	glucoside xylosyltransferase 2		−16	

An additional possibility is that changes in the growth potential of brainstem neurons with axons that project to the spinal cord may be reduced by P28 in opossums. This is an aspect of the failure of neural regeneration in injured adult spinal cord that has been little studied. However, its importance is suggested by the experiments of Kobayashi et al. [120] who showed that infusion of BDNF and NT-4/5 prevented atrophy of rat rubrospinal axons after cervical axotomy. This treatment evoked upregulation of a number of regeneration-associated genes, which they considered correlated with an increased regenerative capacity of axotomized rubrospinal neurons.

### Comparing proteome results from segments of spinal cord rostral and caudal to the transection

The present study reports on the changes in gene and protein expression in the segment of spinal cord rostral to the site of transection of the spinal cord. These changes would be expected to affect centrally projecting neurites from sensory neurons, should they regenerate following injury. There is only limited information about changes in gene expression in the segment of cord caudal to the site of injury in postnatal opossums [24]. That study used a combination of a mouse microarray of genes encoding cytokines and chemokines with some qPCR validation, as discussed above. However, we have previously published a proteomic analysis of the spinal cord segment caudal to the injury in the same animals that have been used in the present study for the segments of cord rostral to the lesion [32].

There were ten proteins in the P7 injured spinal cords that were common to the two segments of cord (Table 6). All but two of these had bands that were decreased in density compared to the controls. Cofilin and peptidylprolyl isomerase A-like proteins were reduced in the rostral spinal cord and increased in the caudal segment. In the P28 injured spinal cord there were fourteen proteins that were common to both segments of cord (Table 7). All but four of the proteins had bands that changed in the same direction in both cord segments, but several of these proteins had bands that increased or decreased in different fractions. As observed at P7, cofilin and peptidylprolyl isomerase a-like decreased in the rostral segment but increased in the caudal segment. Annexin-A2 decreased in the rostral cord but increased in the caudal segment. Tropomyosin on the other hand increased in the rostral segment but decreased caudally. There were more proteins that were identified as changing their regulation only in one segment or the other. These are listed in Tables S7A & S7B for P7+24 h and Tables S7C & S7D for P28+24 h. As we discussed in detail previously [32] some of the observed changes in identified proteins were more likely to be due to post-translational modifications than changes in the expression of their coding genes.

**Table 6:** Proteins that changed level 24*Monodelphis* in segments rostral and caudal to site of transection.

Protein	Rostral SC	Caudal SC
14-3-3	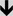	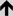 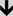
cofilin	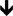	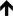
destrin	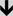	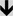
Hemoglobin α	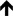 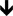	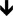
Hemoglobin subunit β M	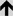	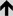 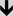
Peptidylprolyl isomerase a-like	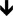	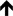
Pyruvate dehydrogenase	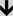	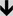
Tubulin α	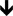	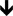
Tubulin β	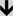	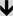
ubiquitin	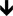	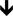

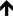
increased band density, 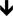 decreased band density. Some proteins showed an increase or decrease in different fractions (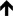 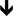).

**Table 7:** Proteins that changed level 24*Monodelphis* in segments rostral and caudal to site of transection.

Protein	Rostral SC	Caudal SC
14-3-3 protein	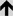	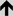 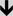
albumin	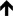	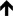 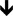
Alpha enolase	□	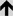 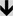
Annexin-a2	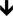	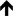
ATP synthase subunit β, mitochondrial	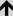	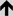 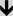
cofilin	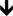	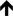
GFAP	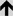	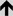
Glyceraldehyde 3 phosphate dehydrogenase	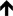 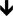	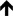 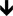
Hemoglobin subunit β M	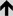 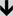	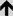 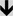
Neurofilament L subunit	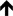	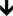
Peptidylprolyl isomerase A-like	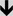	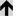
Tropomyosin	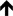	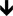
Tubulin α	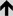 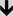	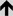 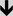
ubiquitin	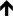 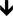	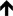

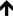
increased band density, 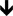 decreased band density. Some proteins showed an increase or decrease in different fractions (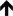 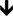).

## Limitations of the Study

This study was restricted to the changes in gene and protein expression that could be identified 24 h after injury at P7 or at P28 in the cord segment rostral to the transection (T10). Further changes in gene and protein expression may well occur at later times following injury as suggested by studies in injured adult spinal cord [16], [103].

This study was initiated early in the adoption of RNA-Seq and involved a small number of samples. Although technical variability is low, a large degree of biological variability was observed, consistent with level of manipulation required in the experimental preparation of samples. This variability is likely to have reduced the number of genes observed to be statistically significantly differentially expressed. This reduced power necessitated the simplification of the analytical design to exclude direct modeling of interaction between development and injury.

In presenting the data we have concentrated on functional categories that previous work in the injured adult spinal cord suggested to be important for the characteristic failure of neurites to grow following injury to the adult spinal cord. This is something that has been known since the time of Ramon y Cajal [121]. Our description has tended to emphasize the genes that showed the largest changes in expression, many of which had not previously been implicated in the failure of neurite outgrowth following spinal cord injury. Since little is known about the functions of some of these genes (e.g., olfactomedin 480 fold; triadin 206 fold and several novel opossum genes, see Table S3) their role in failure of neurite outgrowth is speculative, as was considered in the General Discussion above.

An alternative possibility is that relatively small changes in expression of a large number of key genes may account for the lack of neurite outgrowth. This has been considered in the General Discussion, above.

An important limitation on providing an overall functional view of the large number of expression changes in both genes and proteins is that we were unsuccessful in attempts at pathway analysis, with identified pathways consisting of small number of genes with only one differentially expressed member. We hypothesize that this was primarily because of limitations in the functional annotation of the opossum genome. We also eschewed the use of heat maps for presenting our data in favour of a numerical approach of presenting data in graphs and tables. The visual approach of heat maps puts undue emphasis on expression differences by using colour coding which, although eye catching, may give a misleading impression of the statistical validity of differences in gene expression.

The number of antibodies available that cross react with opossum material is very limited. At this stage it is therefore not possible to define in which cell populations these genes and their protein products are expressed. In future studies it is planned to localize some of the key genes using *in situ* hybridisation.

It may be considered by some that the lack of overlap between the genes we have identified in RNA-Seq analysis and the protein gene products identified in the proteomic part of the study may indicate a fault in the experimental design. There are several reasons for this lack of overlap. The most obvious one is the difference in the sensitivity of the two methods as detection of the protein by even silver staining is several orders of magnitude less than that for RNA. The second main difference is the timing of the extraction protocol—many genes are transcribed much earlier than the translation of their products. Our study was performed at one time point only (24 h post injury). The third, and most biologically relevant possibility is that proteomic analysis identifies not only changes in the concentration levels of individual proteins but also in their post-translational modifications. This would be difficult to detect at the transcriptome level. That this may be the case has been suggested in our earlier proteomic study in which RT-qPCR was used to examine the expression levels of the genes of some of the proteins identified as changing following injury. Many of the identified proteins showed changes in their isoelectric mobility but not in their gene expression [32]. This is important because it indicates that analysis of the transcriptome alone may in fact not provide a comprehensive overall picture of changes occurring in the whole tissue in response to injury.

Despite these limitations the unique nature of these data provide a valuable resource for the investigation of the *a priori* hypotheses of mechanisms of spinal cord regeneration and as a foundation for future studies.

## Conclusions

Morphological repair and functional recovery following spinal cord injury requires a complex but coordinated set of cellular responses; they are particularly complicated because they involve sites close to the injury (local) as well as distant locations such as neuronal cells bodies of axotomised axons. The very large number of genes that changed their expression level significantly following injury, particularly in the more mature spinal cord at P28, is very striking. It reinforces microarray studies of injured adult spinal cord that also show expression changes in a large number of genes [16] indicating just how complicated the response of the spinal cord to injury is. It seems unlikely that the preoccupation of most studies of trying to identify a single gene or protein will provide a therapy for patients with spinal cord injuries, unless key upstream regulatory genes and their proteins can be identified.

In the present study we report on the changes detected in transcript and protein expression that follow complete spinal cord transection at two different ages (P7 and P28) in postnatal *Monodelphis domestica*. P7 pups were chosen because at this age the animals show substantial axon growth across the lesion. This axon growth is partly regenerative from injured axons and partly due to axon growth that occurs as continued postnatal development of the spinal cord [21]. When these animals reach adulthood their locomotor behavior is essentially normal [25]. P28 animals were chosen for comparison because following a similar complete transection there is no axon growth across the lesion although the animals, when adult, exhibit weight-bearing locomotion [25]. The present study was designed to see whether short-term changes in gene and protein expression that occur after a complete spinal transection at T10 might account for these differences in axon growth and subsequent behaviour. Although some immune/inflammatory genes were highly upregulated (Table S3), comparison of the results presented in this study together with previously published work on *Monodelphis* spinal cord *in vitro* suggests that immunological/inflammatory genes may not be involved in the differential response to primary injury we observe at the two ages. However, as we discuss above, they are more likely to be important in the secondary phase of the process.

From the analysis of known functional groups of genes and proteins presented here we conclude that there appear to be at least three not mutually exclusive general mechanisms that may be involved in the age-related response to injury and in particular the lack of neurite outgrowth following injury at P28:

- Changes in expression of genes and proteins that are known to be involved in axon guidance and inhibition (Table 2). However, these were not significant at P7 and at P28 they were relatively small and many were in opposing directions. One example is two of the key *Rho* activating genes (*Arhgap6*, 3.1 fold; *Arhgef10*, −2.1 fold) that are central to control of many of the downstream neurite inhibitory factors. In addition, almost all of the myelin-associated genes and their proteins were either unchanged or downregulated (Tables 2 and 6) which perhaps makes this explanation less likely.
- Down regulation of essential neurotrophic genes in brain stem neurons with axonal projections to the spinal cord. This will be the subject of a separate study (Saunders et al., in preparation).
- Major changes in expression of a few genes at P28 that produce extracellular matrix proteins (Table 3). Most of these have not previously been described in studies of adult spinal cord injury, but they are members of gene families that have been implicated. These seem to be plausible candidates to explain the failure of neurite growth following injury that will be worthy of further study.

This study provides a large database of changes in expression of genes and proteins at 24 h after spinal cord injury at two ages in postnatal *Monodelphis*. We have provided a detailed analysis based on functional groups thought to be important in the response of the spinal cord to injury. There were several novel genes that showed strikingly large changes in expression following injury at P28. Their identity and function will need to be determined before their roles in the response to injury can be considered.

## Supporting Information

Table S1 **Data used for analysis of gene expression changes 24 h following spinal cord transection at P7.** Gene symbols, description and ENSEMBL ID shown. Log2FC = Log_2_ fold change. These values were derived by comparing expression at P7+24 h with P8 control. See Methods for details of statistical analysis used. Updated 13 May 2013.
(XLSX)

Table S2 **Data used for analysis of gene expression changes 24 h following spinal cord transection at P28.** Gene symbols, description and ENSEMBL ID shown. Log2FC = Log_2_ fold change. These values were derived by comparing expression at P28+24 h with P29 control. See Methods for details of statistical analysis used. Updated 13 May 2014.
(XLS)

Table S3 **Gene expression P28+24 h following spinal cord transection.** Genes categorized by function with their ID and description included. FC = Fold Change compared to P29 controls. Updated 13 May 2014.
(XLSX)

Table S4
**Proteins that changed expression level 24 h following spinal cord injury at P7 in** ***Monodelphis domestica*** **.** Arrows indicate direction of change in gel band density (upregulation 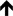
or downregulation 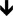). Relative change is densitometry value of gel band at P7+24 h compared to P8 control. Gene name symbol or provisional ID in genome and protein function are included.(DOCX)

Table S5
**Proteins that changed expression level 24 h following spinal cord injury at P28 in** ***Monodelphis domestica*** **.** Arrows indicate direction of change in gel band density (upregulation 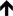 or downregulation 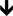). Relative change is densitometry value of gel band at P28+24 h compared to P29 control. Gene name, symbol or provisional ID in genome and protein function are included.(DOCX)

Table S6 **Proteomic results of identified proteins in different fractions and bands compared with expression of their respective genes in the transcriptome.** ***Column A*** shows fraction in which protein was identified, ***Column B*** shows band in gel in which protein was identified. Note that some proteins were identified in more than one fraction or band. ***Column C*** is ENSEMBL protein ID, where known. ***Column D*** is protein name. ***Column E*** and ***F*** show whether protein band density was increased ≥0.5 compared to age-matched controls 24 h following spinal cord injury at P7 or P28. ***Column G*** shows corresponding gene symbol or name. ***Column H*** shows ENSEMBL gene ID. ***Column I*** and ***J*** show gene expression level 24 h following injury at P7 or P28. Note the relative lack of correlation between identified changes in the transcriptome and the proteome. Updated 13 May 2014
(XLSX)

Table S7
**A. Proteins that were identified as changing expression level in the spinal cord** ***rostral*** **but not caudal to the site of transection 24 h after injury at P7.** 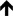 increased band density, 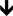 decreased band density. Some proteins showed an increase or decrease in different fractions (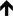 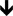). **B. Proteins that were identified as changing in expression level in the spinal cord** ***caudal*** **but not rostral to the site of transection 24 h after injury at P7.** 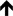 increased band density, 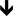 decreased band density. Some proteins showed an increase or decrease in different fractions (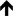 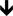). **C**. *Monodelphis* spinal cord. Proteins that were identified in the spinal cord *rostral* but not caudal to the site of transection 24 h after injury at P28. 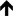 increased band density, 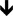 decreased band density. Some proteins showed an increase or decrease in different fractions (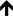 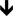). **D**: *Monodelphis* spinal cord. Proteins that were identified in the spinal cord *caudal* but not rostral to the site of transection 24 h after injury at P28. 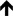
increased band density, 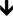
decreased band density. Some proteins showed an increase or decrease in different fractions (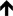 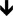).(DOCX)
