# Supplementary material for: Age-Dependent Transcriptome and Proteome Following Transection of Neonatal Spinal Cord of Monodelphis domestica (South American Grey Short-Tailed Opossum)
Source: PLoS One. 2014 Jun 10;9(6):e99080. doi: 10.1371/journal.pone.0099080 (PMC4051688; doi:10.1371/journal.pone.0099080)
Supplement: Table S4 — Proteins that changed expression level 24 h following spinal cord injury at P7 in Monodelphis domestica . Arrows indicate direction of change in gel band density (upregulation or downregulation ). Relative change is densitometry value of gel band at P7+24 h compared to P8 control. Gene name symbol or provisional ID in genome and protein function are included. (DOCX) [file pone.0099080.s004.docx]

**Supplementary Table S4. Proteins that changed expression level 24h following spinal cord injury at P7 in *Monodelphis domestica.*** Arrows indicate direction of change in gel band density (upregulation 🡹 or downregulation 🡻). Relative change is densitometry value of gel band at P7+24h compared to P8 control. Gene name symbol or provisional ID in genome and protein function are included.

| PROTEIN NAME | CHANGE | RELATIVE CHANGE | GENE | FUNCTION |
| --- | --- | --- | --- | --- |
| BLOOD-RELATED | | | | |
| Hemoglobin α | 🡹🡻 | 2.06 and 0.35 | *Hba1* | carriage of O_2_ in blood |
| Hemoglobin subunit β-M | 🡹 | 2.06 | *Loc100019389* | carriage of O_2_ in blood |
| NEURITE INHIBITION, GUIDANCE & EXTRACELLULAR PROTEINS | | | | |
| α-tubulin | 🡻 | 0.5 | *Tuba1b* | neurogenesis & axon guidance |
| β-tubulin | 🡻 | 0.5 | *Tubb3* | neurogenesis & axon guidance |
| Brain specific protein (Tubulin polymerization-promoting protein family member 3) | 🡻 | 0.5 | *Tppp3* | tubulin polymerization-promoting protein family member 3 |
| Myelin basic protein | 🡹 | 2.06 | *Mbp* | inhibition of neurite outgrowth |
| Ubiquinol-cytochrome c reductase core protein II | 🡻 | 0.5 | *Uqcrc2* | interacts with nogo |
| APOPTOSIS AND UBIQUITIN RELATED | | | | |
| Proliferation associated gene (pag)  peroxiredoxin1 | 🡻 | 0.49 | *Prdx1* | antioxidant protective role |
| Peroxiredoxin-2 | 🡻 | 0.49 | *Prdx2* | antioxidant protective role |
| Proteasome (prosome, macropain) subunit β type 1 | 🡻 |  | *Psmb1* | immunoproteasome, processin of class I MHC peptides |
| Ubiquitin c | 🡻 | 0.49 | *Ubc* | Polyubiquitin precursor |
| Ubiquitin A-52 residue ribosomal protein fusion product | 🡻 | 0.34 | *Uba52* | fusion protein |
| Ubiquitin specific peptidase 30 phosphoglycerate kinase | 🡻 | 0.5 | *Usp30* | novel mitochondrial deubiquitinating (DUB) enzyme |
| SYNAPSES, NEURAL RECEPTORS & CHANNELS | | | | |
| β-actin isoform 1 | 🡻 | 0.5 | *TGM1-transglutaminase* | not previously identified in spinal cord |
| Cofilin-2-like | 🡻 | 0.5 | *Loc100016593* | Rho-induced reorganization of actin cytoskeleton |
| Cytoplasmic dynein light chain 1 | 🡻 | 0.1 | *Dynll1* | intracellular transport & motility |
| Destrin | 🡻 | 0.5 | *Dstn* | actin-binding proteins, ADF family |
| Dynein light chain lc8-type 2 | 🡻 | 0.1 | *Dynll2* | intracellular transport & motility |
| 14-3-3 ε | 🡻 | 0.32 | *Ywhae* | tyrosine 3-monooxygenase /tryptophan 5-monooxygenase activation |
| Valosin | 🡹 | 2.75 | *Vcp* | vesicle transport & fusion |
| DEVELOPMENT & STRUCTURE | | | | |
| Glial fibrillary acidic protein | 🡻 | 0.5 | *Gfap* | intermediate filament in astrocytes |
| KIAA0120, transgelin 2 | 🡻 | 0.5 | *Tagln2* | marker of differentiated smooth muscle |
| Peptidylprolyl isomerase A-like | 🡻 | 0.5 | *Ppial4a* | catalyzes the cis-trans isomerization of proline imidic peptide bonds in oligopeptides |
| Phosphatidylethanolamine-binding protein 1 | 🡻 | 0.5 | *Pebp1* | interacts with MAP2K1, C-Raf and MAPK1 |
| METABOLIC ASSOCIATED | | | | |
| 6-phosphogluconate dehydrogenase | 🡻 | 0.45 | [*Pgd*](http://www.ncbi.nlm.nih.gov/gene/5226) | part of pentose phosphate pathway |
| ATP synthase subunit β | 🡻 | 0.5 | [*Atp5b*](http://www.ncbi.nlm.nih.gov/gene/506) | catalyzes ATP synthesis, |
| Fructose bisphosphate aldolase C | 🡻 | 0.16 | [*Aldoc*](http://www.ncbi.nlm.nih.gov/gene/230) | glycolytic enzyme |
| Triosephosphate isomerase | 🡻 | 0.17 | [*Tpi1*](http://www.ncbi.nlm.nih.gov/gene/7167) | catalyzes isomerization G3P & DHAP glycolysis & gluconeogenesis |
| Pyruvate dehydrogenase (lipoamide) (EC 1.2.4.1) alpha chain precursor | 🡻 | 0.16 | [*Pdha1*](http://www.ncbi.nlm.nih.gov/gene/5160) | glycolysis & tricarboxylic acid cycle |
| STRESS RESPONSE | | | | |
| Cold inducible RNA binding protein | 🡻 | 0.49 | [*Cirbp*](http://www.ncbi.nlm.nih.gov/gene/1153) | controls cellular response to stress |
| Heat shock protein 90B1 | 🡹 | 1.5 | *Hsp90b1* | stabilizing and folding proteins |
