# Supplementary material for: Age-Dependent Transcriptome and Proteome Following Transection of Neonatal Spinal Cord of Monodelphis domestica (South American Grey Short-Tailed Opossum)
Source: PLoS One. 2014 Jun 10;9(6):e99080. doi: 10.1371/journal.pone.0099080 (PMC4051688; doi:10.1371/journal.pone.0099080)
Supplement: Table S5 — Proteins that changed expression level 24 h following spinal cord injury at P28 in Monodelphis domestica . Arrows indicate direction of change in gel band density (upregulation or downregulation ). Relative change is densitometry value of gel band at P28+24 h compared to P29 control. Gene name, symbol or provisional ID in genome and protein function are included. (DOCX) [file pone.0099080.s005.docx]

**Supplementary Table S5. Proteins that changed expression level 24h following spinal cord injury at P28 in *Monodelphis domestica*.** Arrows indicate direction of change in gel band density (upregulation 🡹 or downregulation 🡻). Relative change is densitometry value of gel band at P28+24h compared to P29 control. Gene name, symbol or provisional ID in genome and protein function are included.

| PROTEIN NAME | CHANGE | RELATIVE CHANGE | GENE | FUNCTION |
| --- | --- | --- | --- | --- |
| IMMUNE/INFLAMMATORY | | | | |
| Annexin A2 | 🡻 | 0.45 | *Anxa2* | recruitment & activation of immune cells |
| Immunoglobulin lambda-like polypeptide 5-like | 🡹 | 1.46 | *Loc100617965* | not known |
| Melanocortin 1 receptor | 🡻 | 0.5 | *Mc1r* | ligands exert anti-inflammatory and immunomodulatory effects |
| BLOOD-RELATED | | | | |
| Hemoglobin α | 🡻 | 0.5 | *Hba1* | carriage of O_2_ in blood |
| Hemoglobin subunit β-M | 🡹🡻 | 5.5, 1.91, 0.3, 0.5 | *Loc100019389* | carriage of O_2_ in blood |
| Albumin | 🡹 | 3.03 | *Alb* | carrier protein |
| NADPH-flavin reductase | 🡹 | 2.78 | *Blvrb* | heme metabolism |
| NEURITE INHIBITION, GUIDANCE & EXTRACELLULAR PROTEINS | | | | |
| α-tubulin | 🡹🡻 | 0.5 | *Tuba1b* | neurogenesis, axon guidance & maintenance |
| β-tubulin | 🡹🡻 | 1.98 and 0.5 | *Tubb3* | neurogenesis, axon guidance & maintenance |
| Elongation factor 2 isoform 1 | 🡻 | 0.5 | *Eef2* | regulates neurite outgrowth |
| Lectin, galactoside-binding, soluble, 1 (galactin1) | 🡹 | 1.91 | *Lgals1* | regeneration of various spinal cord tracts |
| Myelin basic protein | 🡻 | 0.5 | *Mbp* | inhibition of neurite outgrowth |
| RhoGDI-1 | 🡹 | 5.4 | *Arhgdia* | Rho GTPase activating protein |
| 14-3-3 σ | 🡹 | 1.46 | *Sfn* | modulates production of extracellular matrix proteins. Stimulates MMP1 |
| APOPTOSIS & UBIQUITIN | | | | |
| NEDD8-conjugating enzyme UBC12 | 🡹 | 2.78 | *Ubc12* | NEDD8 conjugation to cell proteins |
| Peroxiredoxin-2 | 🡹 | 3.23 | *Prdx2* | antioxidant protective role |
| Peroxiredoxin-6 | 🡹🡻 | 1.73 and 0.5 | *Prdx6* | redox regulation |
| Ubiquitin c | 🡹🡻 | 2.1 and 0.44 | *Ubc* | polyubiquitin precursor |
| Ubiquitin A-52 residue ribosomal protein fusion product | 🡹🡻 | 1.64 and 0.23 | *Uba52* | fusion protein |
| SYNAPSES & NEURAL RECEPTORS & CHANNELS | | | | |
| 14-3-3 ε | 🡹 | 1.46 | *Ywhae* | tyrosine 3-monooxygenase /tryptophan 5-monooxygenase activation, signal transduction |
| 14-3-3 γ | 🡹 | 1.46 | *Ywhag* | tyrosine 3-monooxygenase /tryptophan 5-monooxygenase activation, signal transduction |
| 14-3-3 ζ | 🡹 | 5.4 | *Ywhaz* | tyrosine 3-monooxygenase /tryptophan 5-monooxygenase activation, signal transduction |
| Calbindin 2 | 🡹 | 5.4 | *Calb2* | intracellular calcium-binding proteinmodulator of neuronal excitability |
| Cofilin-2-like | 🡹 | 0.5 | *Loc100016593* | Rho-induced reorganization of actin cytoskeleton |
| Cytoplasmic dynein light chain 1 | 🡹🡻 | 1.62 and 0.04 | *Dynll1* | intracellular transport and motility |
| Dynein light chain LC8 type 2 | 🡹🡻 | 1.62 and 0.04 | *Dynll2* | intracellular transport & motility |
| Lasp-1 | 🡹 | 0.45 | *Lasp1* | Actin binding |
| Neurocalcin delta | 🡹 | 2.05 | *Ncald* | neuronal calcium sensor, modulates neurotransmitter release |
| GTP-binding protein REM 1-likebinding 1 | 🡹 | 2.05 | *Rem1* | reorganization of actin cytoskeleton |
| Tropomyosin, γ isoform | 🡹 | 5.4 and 1.5 | *Tpm3* | actin filament stability |
| Valosin | 🡹 | 2.8 | *Vcp* | vesicle transport & fusion |
| Visinin like-1 | 🡹 | 3.23 and 2.05 | *Vsnl1* | neuronal calcium sensor |
| pyruvate dehydrogenase | 🡹 | 2.46 | *Pdha1* | synaptosomal complex |
| DEVELOPMENT & STRUCTURE | | | | |
| Parkinson protein 7 | 🡹 | 2.78 | *Park7* | protects neurons against oxidative stress & cell death |
| Glial fibrillary acidic protein | 🡹 | 1.98 | *Gfap* | intermediate filament in astrocytes |
| Neurofilament L-subunit | 🡹 | 1.51 | *Dock5* | axoskeleton & transport |
| Nucleophosmin | 🡹 | 2.46 | *Npm1* | regulates ARF/p53 pathway |
| Peptidylprolyl isomerase 4A-like | 🡻 | 0.3 and 0.5 | *Ppial4a* | catalyzes cis-trans isom of proline imidic peptide bonds |
| Protein phosphatase 2 (formerly 2A), regulatory subunit B | 🡻 | 0.5 | *Ppp2r2b* | negative cell growth and division |
| METABOLIC ASSOCIATED | | | | |
| 6-phosphogluconate dehydrogenase | 🡻 | 0.38 | *Pgd* | pentose phosphate pathway |
| Acetyl coenzyme A acetyltransferase 2 | 🡻 | 0.5 | *Acat2* | lipid metabolism |
| α enolase (2-phospho-D-glycerate hydrolyase) | 🡻 | 0.5 | *Eno1* | glycolysis, amino acid synthesis |
| ATP synthase subunit beta, mitochondrial | 🡹 | 1.98 | *Atp5b* | catalyzes ATP synthesis, |
| Glyceraldehyde-3-phosphate dehydrogenase | 🡹🡻 | 2.5 and 0.5 | *Gapdh* | carbohydrate metabolism |
| Fructose bisphosphate aldolase C | 🡹🡻 | 0.5 | *Aldoc* | glycolytic enzyme |
| Gluthathione transferase M3 | 🡻 | 0.5 | *Gstm3* | gluthathione conjugation |
| Myristoylated alanine rich C-kinase substrate | 🡹🡻 | Control only and 1.46 | *Marcks* | membrane trafficking |
| Pyruvate dehydrogenase (lipoamide) (EC 1.2.4.1) alpha chain precursor | 🡻 | 0.5 | *Pdha1* | glycolysis and tricarboxylic acid cycle |
| Triosphosphate isomerase | 🡹🡻 | 1.73 and 0.1 | *Tpi1* | catalyzes isomerization of G3P & DHAP in glycolysis & gluconeogenesis |
| STRESS RESPONSE | | | | |
| Heat shock protein 90 | 🡹🡻 | 4.3 and 1.5 | *Hsp90aa1* | heat shock protein 90 |
| Glucose regulated protein heat shock 70kDa protein 5 | 🡻 | 1.98 | *Hspa5* | ER stress-induced apoptosis response protein |
| Heat shock protein 84b | 🡹 | 4.3 | *Hsp90ab1* | signal transduction, protein folding, degradation |
| Heat shock protein 90B1 | 🡹 | 4.3 | *Hsp90b1* | stabilizing and folding proteins |
| Novel Heat shock protein | 🡻 | 0.31 | *Loc100027477* | heat shock cognate 71 kDa protein-like |
| Heat shock protein | 🡻 | 0.31 | *Hspa8* | heat shock cognate 71 kDa protein-like |
| UNKNOWN | | | | |
| Chain A crystal structure of human translationally controlled tumour associated protein | 🡹 | 3.23 | *Loc100018984* | endocytosis |
