# Supplementary material for: Age-Dependent Transcriptome and Proteome Following Transection of Neonatal Spinal Cord of Monodelphis domestica (South American Grey Short-Tailed Opossum)
Source: PLoS One. 2014 Jun 10;9(6):e99080. doi: 10.1371/journal.pone.0099080 (PMC4051688; doi:10.1371/journal.pone.0099080)
Supplement: Table S7 — A. Proteins that were identified as changing expression level in the spinal cord rostral but not caudal to the site of transection 24 h after injury at P7. increased band density, decreased band density. Some proteins showed an increase or decrease in different fractions ( ). B. Proteins that were identified as changing in expression level in the spinal cord caudal but not rostral to the site of transection 24 h after injury at P7. increased band density, decreased band density. Some proteins showed an increase or decrease in different fractions ( ). C. Monodelphis spinal cord. Proteins that were identified in the spinal cord rostral but not caudal to the site of transection 24 h after injury at P28. increased band density, decreased band density. Some proteins showed an increase or decrease in different fractions ( ). D: Monodelphis spinal cord. Proteins that were identified in the spinal cord caudal but not rostral to the site of transection 24 h after injury at P28. increased band density, decreased band density. Some proteins showed an increase or decrease in different fractions ( ). (DOCX) [file pone.0099080.s007.docx]

**Supplementary Table S7A. Proteins that were identified as changing expression level in the spinal cord *rostral* but not caudal to the site of transection 24h after injury at P7.** 🡹 increased band density, 🡻 decreased band density. Some proteins showed an increase or decrease in different fractions (🡹🡻).

| 6-phosphogluconate dehydrogenase | 🡻 | Peroxiredoxin-2 (thioredoxin peroxidase 1) | 🡻 |
| --- | --- | --- | --- |
| ATP synthase subunit β, mitochondrial | 🡻 | Phosphatidylethanolamine-binding protein 1 (PEBP-1) | 🡻 |
| Brain specific protein | 🡻 | Precursor polypeptide (AA-21 to 782) | 🡻 |
| Cold inducible RNA binding protein | 🡻 | Proliferation associated gene (pag) | 🡻 |
| Cytoplasmic dynein light chain 1 | 🡻 | Proteasome (prosome, macropain) subunit, β type 1 | 🡻 |
| Dynein, light chain, LC8-type 2 | 🡻 | Ubiquitin A-52 residue ribosomal protein fusion | 🡻 |
| Fructose bisphosphate aldolase C | 🡻 | Ubiquitin specific peptidase 30 phosphoglycerate kinase | 🡻 |
| Glial fibrillary acidic protein, astrocyte | 🡻 | UQCRC2 protein | 🡻 |
| KIAA0120 | 🡻 | Valosin | 🡹 |
| Myelin basic protein | 🡹 |  |  |

**Supplementary Table S7B. Proteins that were identified as changing in expression level in the spinal cord *caudal* but not rostral to the site of transection 24h after injury at P7.** 🡹 increased band density, 🡻 decreased band density. Some proteins showed an increase or decrease in different fractions (🡹🡻).

| Albumin | 🡹🡻 | General transcription factor II I isoform 4 | 🡹 | Lactoglobulin |  |
| --- | --- | --- | --- | --- | --- |
| α enolase | 🡻 | Glucose regulated protein 78 | 🡹🡻 | Malate dehydrogenase | 🡻 |
| ATP synthase α subunit | 🡻 | Glyceraldehyde 3 phosphate dehydrogenase | 🡹🡻 | Peptidylprolyl isomerase B | 🡻 |
| Casein α 1 | 🡻 | Hemoglobin embryonic β chain | 🡻 | Pol polyprotein | 🡹🡻 |
| Chaperonin containing t-complex polypeptide 1, β subunit | 🡻 | Heterogeneous nuclear ribonucleoprotein A2/B1 | 🡹 | Profilin | 🡻 |
| crmp2a | 🡻 | Heat shock protein 1 | **🡻** | Transketolase | 🡻 |
| dpysl3 | 🡻 | Heat shock protein 90 | 🡹🡻 | Tropomyosin 2 | 🡹 |
| Elongation factor 1 | 🡻 | Heat shock protein 60 | 🡹 | Voltage dependent anion selective channel protein 1 | 🡻 |
| Fatty acid binding protein (brain type) | **🡻** | Internexin neuronal intermediate filament-α | 🡻 | Voltage dependent anion channel 3 | 🡻 |
| gdp dissociation inhibitor 1 | 🡻 | Lactate dehydrogenase | 🡻 |  |  |

**Supplementary Table S7C:** *Monodelphis* spinal cord. Proteins that were identified in the spinal cord *rostral* but not caudal to the site of transection 24h after injury at P28. 🡹 increased band density, 🡻 decreased band density. Some proteins showed an increase or decrease in different fractions (🡹🡻).

| Stratifin 14-3-3 protein sigma | 🡹 | Cytoplasmic dynein light chain 1 | 🡹🡻 | LASP-1 | 🡻 |
| --- | --- | --- | --- | --- | --- |
| 6-phosphogluconate dehydrogenase | **🡻** | Dynein, light chain, LC8-type 2 | 🡹🡻 | Myelin basic protein | 🡻 |
| AC39/physophilin | **🡹** | Elongation factor 2 isoform 1 | 🡹 | Myristoylated alanine rich C-kinase substrate (MARCKS) | 🡹🡻 |
| Acetyl coenzyme A acetyletransferase 2 | **🡻** | Fructose bisphosphate aldolase C | 🡹🡻 | NADPH-flavin reductase | 🡹 |
| Aldolase C, fructose bisphosphate | **🡹🡻** | Glucose regulated protein heat shock 70kDa protein 5 | 🡻 | NEDD8-conjugating enzyme UBC12 | 🡹 |
| Calretinin | **🡹** | Glutathione transferase M3 | 🡻 | neurocalcin | 🡹 |
| CAP1 protein | **🡹** | Heat shock protein 84b | 🡹 | Non-selenium glutathione phospholipid hydroperoxide peroxidase | 🡹🡻 |
| Chain A, crystal structure of human translationally controlled tumour associated protein | **🡹** | Hemoglobin α | 🡻 | Nucleophosmin | 🡹 |
| Chain B, X-ray crystal structure of C2s human galectin-1 complexed with galactose | **🡹** | Heat shock protein 90 | 🡹 | Peroxiredoxin-2 (thioredoxin peroxidase 1) | 🡹 |

**Supplementary Table S7D:** *Monodelphis* spinal cord. Proteins that were identified in the spinal cord *caudal* but not rostral to the site of transection 24h after injury at P28.🡹increased band density, 🡻decreased band density. Some proteins showed an increase or decrease in different fractions (🡹🡻).

| ATP synthase α subunit | 🡻 | Malate dehydrogenase 2, NAD (mitochondrial) | 🡹🡻 |
| --- | --- | --- | --- |
| cpn10 | **🡻** | Peptidylprolyl isomerase B | 🡹 |
| crmp2a | **🡹** | Pol polyprotein | 🡹 |
| Elongation factor 1 | **🡻** | Profilin | 🡻 |
| Fatty acid binding protein (heart type) | **🡹🡻** | Pyruvate kinase (muscle) | 🡹 |
| Heterogeneous nuclear ribonucleoprotein A2/B1 | **🡹🡻** | Voltage dependent anion selective channel protein 1 | 🡹🡻 |
| Heat shock protein 1β | **🡹🡻** | Voltage dependent anion channel 3 | 🡹 |
| Lactate dehydrogenase | **🡻** |  |  |
